# Genomic Determinants of Induction Failure and Persistent Prognostic Risk of *FLT3*-ITD in Pediatric Acute Myeloid Leukemia

**DOI:** 10.3390/cancers18203280

**Published:** 2026-10-12

**Authors:** Rongqiu Liu, Yinghui Cui, Lan Huang, Xianmin Guan, Yali Shen, Ying Dou, Xianhao Wen, Yuxia Guo, Haiyan Liu, Xinyuan Yao, Yan Xiang, Xing Zeng, Qiao Liu, Jie Yu

**Affiliations:** Department of Hematology and Oncology, Children’s Hospital of Chongqing Medical University, National Clinical Research Center for Children and Adolescents’ Health and Diseases, Ministry of Education Key Laboratory of Child Development and Disorders, Intelligent Application of Big Data in Pediatrics Engineering Research Center of Chongqing Education Commission of China, Chongqing 400014, China

**Keywords:** pediatric acute myeloid leukemia, induction failure, *FLT3*-ITD, genomic landscape, risk stratification

## Abstract

Background: Induction failure (IF) after standard induction chemotherapy is associated with poor outcomes in pediatric acute myeloid leukemia (pAML); however, whether established molecular markers such as *FLT3*-ITD retain prognostic significance across induction response states remains incompletely understood. We characterized the genomic landscape of IF and evaluated the prognostic significance of *FLT3*-ITD according to induction response. Methods: This multi-cohort study integrated a TARGET discovery cohort (*n* = 1979) and an independent cohort from the Children’s Hospital of Chongqing Medical University (CHCMU; *n* = 98). Clinical characteristics, somatic mutations, fusion profiles, and gene–gene interaction patterns were compared between patients with and without IF. Multivariable logistic regression identified genomic factors associated with IF, and survival analyses evaluated the prognostic impact of *FLT3*-ITD according to induction response. Results: IF was associated with inferior OS (overall survival) (pooled HR = 3.24, 95% CI: 2.66–3.95). *FLT3* mutations were consistently enriched in IF patients, whereas *KIT* mutations and favorable core-binding factor fusions were more frequent among non-IF patients. *NUP98*-*NSD1* fusion was strongly enriched in IF patients in the TARGET cohort. Gene interaction analyses revealed reduced recurrent co-mutation patterns among IF patients. *FLT3* mutation independently and reproducibly associated with IF. Among patients achieving induction remission, *FLT3*-ITD remained associated with inferior OS (HR = 1.34, 95% CI: 1.06–1.69, *p* = 0.014), whereas no statistically significant additional prognostic effect was detected among patients with IF. Conclusions: This study characterizes the genomic landscape of IF in pAML and identifies *FLT3* mutation as a reproducible marker of induction resistance. Achieving induction remission does not eliminate the adverse prognostic significance of *FLT3*-ITD, supporting the integration of molecular risk and treatment response in subsequent risk stratification.

## Simple Summary

Induction failure (IF), the persistence of leukemia after initial chemotherapy, is associated with poor survival in children with acute myeloid leukemia (AML), yet its genetic basis remains incompletely understood. This study compared the genetic profiles of children with and without IF using two independent patient cohorts. We found that mutations in the *FLT3* gene were consistently more common in children who failed induction, and that the *NUP98*-*NSD1* fusion was strongly enriched in this group. Importantly, the high-risk marker *FLT3*-ITD remained associated with worse survival even in children who successfully achieved remission, showing that reaching remission does not eliminate this risk. These findings highlight the genetic heterogeneity of IF and support combining genetic information with treatment response to guide more individualized risk assessment and treatment decisions.

## 1. Introduction

Pediatric acute myeloid leukemia (pAML) accounts for approximately 20% of childhood acute leukemias and remains a major cause of leukemia-related mortality in children [1,2,3,4]. Although risk-adapted chemotherapy and hematopoietic stem cell transplantation (HSCT) have improved outcomes, a substantial proportion of patients still fail to achieve remission or relapse after therapy [5,6,7,8]. Among these adverse outcomes, induction failure (IF), defined by persistent bone marrow (BM) blasts or extramedullary disease after two cycles of induction chemotherapy [9,10], occurs in approximately 15% of pAML patients [11], and represents one of the strongest indicators of treatment resistance, predicting extremely poor clinical outcomes [12].

The biological basis of IF in pAML remains incompletely understood. Because induction response is determined after treatment has begun, pretreatment clinical and genomic risk factors alone may inadequately account for the biological heterogeneity underlying primary chemotherapy resistance. Current treatment strategies increasingly incorporate targeted therapies for selected molecular subgroups, including *FLT3*-directed agents and other targeted approaches [10]. Nevertheless, IF remains a clinically important manifestation of treatment resistance, highlighting the need for better identification of patients at risk of failing initial therapy.

Unlike adult AML, pAML exhibits a distinct genomic landscape characterized by fewer recurrent driver mutations but frequent structural alterations and fusion events [13]. Although current risk stratification systems incorporate clinical, cytogenetic, and molecular features, they primarily rely on pretreatment characteristics and may not fully capture intrinsic resistance to induction therapy. Comprehensive genomic approaches may provide additional insights into the biological basis of IF [14,15,16].

Previous large-scale genomic studies, including the TARGET initiative, have substantially advanced molecular classification and prognostic assessment in pAML. However, most studies have focused on disease subtypes, overall genomic architecture, or long-term outcomes rather than specifically investigating treatment response phenotypes [17]. Moreover, whether genomic predictors identified in Western populations are applicable to East Asian patients remains uncertain [13,18,19]. Comprehensive analyses integrating somatic mutations, fusion landscapes, and gene–gene interaction patterns across independent populations are still lacking [20,21].

Among recurrent molecular abnormalities in pAML, *FLT3* alterations, particularly *FLT3*-internal tandem duplication (*FLT3*-ITD), represent one of the most clinically relevant high-risk biomarkers and have been associated with increased relapse risk and inferior survival [22,23]. However, whether the prognostic significance of *FLT3*-ITD persists after achievement of induction remission remains unclear.

To address these knowledge gaps, we performed an integrative genomic analysis using a large TARGET discovery cohort and an independent CHCMU validation cohort. We systematically characterized genomic differences between IF and non-IF patients, including recurrent mutations, fusion events, and gene–gene interaction patterns. Furthermore, we identified and validated genomic factors associated with IF and investigated whether *FLT3*-ITD retains its adverse prognostic significance among patients achieving induction remission. Our study provides a comprehensive framework for understanding the molecular basis of IF and supports the integration of dynamic treatment response with genomic profiling for improved risk stratification and identification of patients with primary chemotherapy resistance.

## 2. Materials and Methods

### 2.1. Study Population and Datasets

This study adopted a two-cohort observational design including a public discovery cohort and an independent validation cohort to investigate clinical and genomic determinants of IF in pAML. The discovery cohort was derived from the Therapeutically Applicable Research to Generate Effective Treatments (TARGET) AML program. Clinical and genomic data were obtained from the Genomic Data Commons (GDC) portal (data frozen on 25 October 2024). After excluding patients with unknown induction response, pre-remission death, and FAB-M3 subtype, a total of 1979 patients were enrolled for clinical and survival analysis, among whom 659 patients with available somatic mutation data were included for genomic analyses and identification of genomic predictors of IF.

The validation cohort consisted of 98 consecutive pAML patients diagnosed and treated at the Children’s Hospital of Chongqing Medical University (CHCMU) from March 2019 to October 2023. Patients with FAB-M3 subtype or incomplete induction treatment were excluded. All enrolled patients completed standard dual-cycle induction therapy with complete clinical and genomic data available.

**Figure 1:**
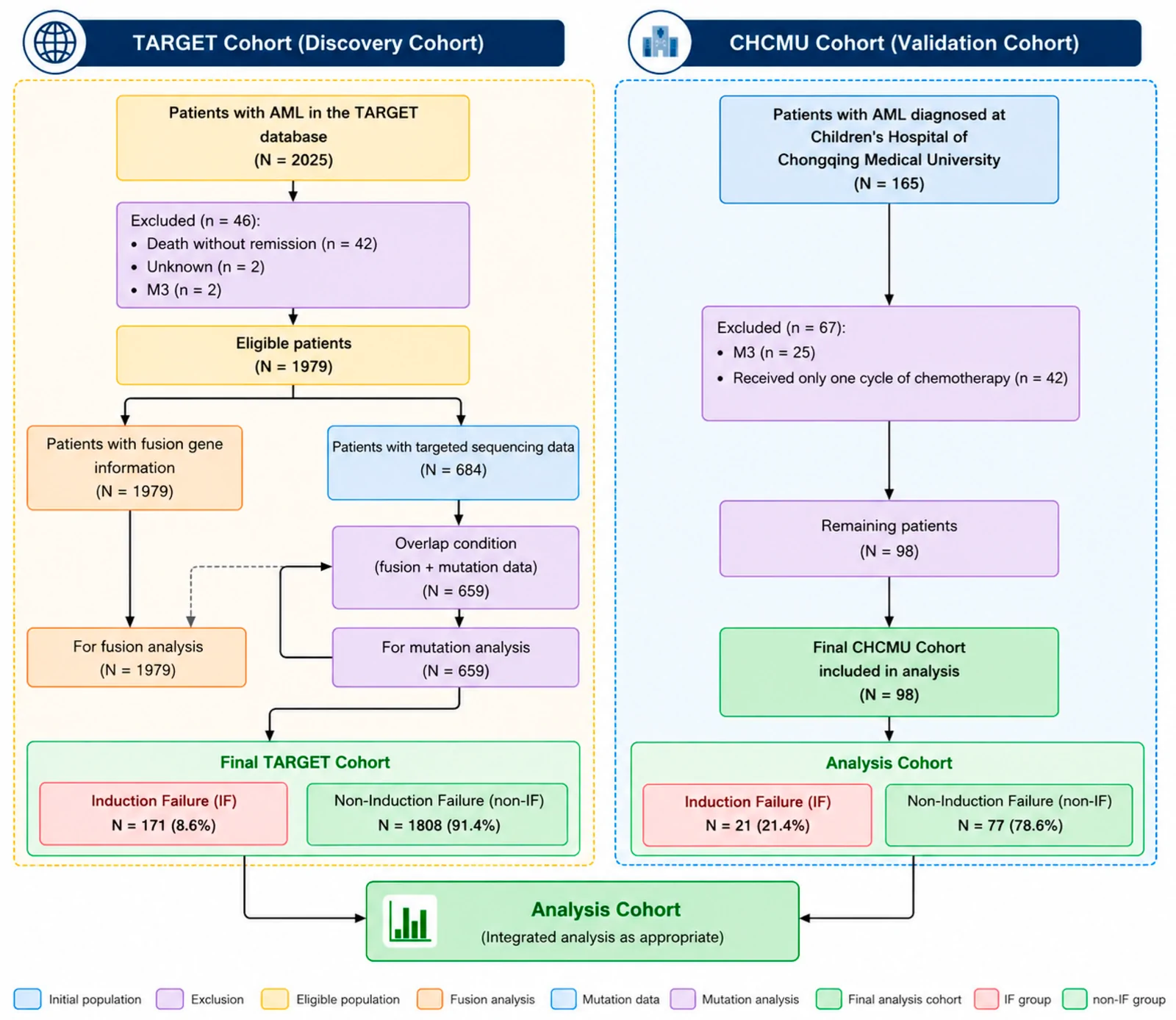
Study design and cohort selection. Overview of the study workflow, including patient selection, clinical characterization, genomic profiling, and validation analyses in the TARGET discovery cohort and CHCMU validation cohort.

The final TARGET cohort included 171 IF and 1808 non-IF patients, whereas the CHCMU cohort included 21 IF and 77 non-IF patients. A detailed flowchart of the study design and patient screening process is presented in Figure 1.

### 2.2. Clinical Endpoints and Baseline Variables

IF was defined specifically as ≥5% bone marrow (BM) blasts or extramedullary disease at the end of induction course 2 [9]. Overall survival (OS) was defined as the time from diagnosis to death or last follow-up. Event-free survival (EFS) was defined as the time from diagnosis to IF, relapse, or death, whichever occurred first. Patients who developed IF were counted as EFS events at the date on which IF was documented at the end of induction course 2, rather than at the time of diagnosis. EFS and OS were analyzed as separate endpoints with distinct event definitions; being counted as an EFS event did not censor or otherwise remove a patient from the OS analysis, in which IF status was evaluated as the exposure of interest. EFS analyses were considered exploratory secondary analyses, with OS serving as the primary endpoint. Baseline clinical variables included age, sex, pretreatment peripheral white blood cell (WBC) count, BM and peripheral blast percentage, FAB subtype, central nervous system involvement, and treatment regimen.

### 2.3. Genomic Profiling and Annotation

Somatic mutation data were obtained from publicly available TARGET AML genomic datasets generated through sequencing platforms including whole-genome sequencing and targeted capture sequencing of recurrent AML-associated genes. Among the 684 cases with multi-platform-verified variants, 659 patients with complete clinical response annotation and somatic mutation data were included in the genomic association analyses [13]. For the CHCMU validation cohort, total RNA was extracted from pretreatment BM mononuclear cells, and RNA quality was assessed before library construction. RNA libraries were subjected to 150-bp paired-end sequencing on the Illumina platform (Illumina, San Diego, CA, USA), with a minimum of 15 Gb of pass-filter data generated per sample. Sequencing reads were aligned to the GRCh37 reference genome using STAR (version 2.7.11). Putative variants were identified using VarScan (version 2.3.9.1) and annotated according to pathogenicity criteria. Gene fusions were detected using Arriba (version 2.4.0) and annotated against multiple fusion databases.

The TARGET and CHCMU cohorts used different sequencing platforms (multi-platform DNA sequencing vs. RNA-seq), which may affect the detection of structural and copy-number alterations. However, within the CHCMU cohort, all 98 samples were processed uniformly with a single library preparation protocol, a single sequencing platform, and a single standardized bioinformatic pipeline, so platform heterogeneity applies only to cross-cohort comparisons. Cross-cohort comparisons were therefore restricted to genes robustly detectable in both datasets, and negative CHCMU findings were conservatively interpreted.

CHCMU variants were classified according to AMP/ASCO/CAP guidelines and Chinese expert consensus criteria [24]. Only Levels 1 (clearly pathogenic) and 2 (potentially pathogenic) were used for co-mutation and gene interaction analyses to minimize false-positive signals; Level 3 variants were excluded from these analyses but were descriptively summarized. All detected mutations (Levels 1–3) were included as candidate predictors in logistic regression analyses to maximize sensitivity for identifying IF-associated genomic features. TARGET variants required no additional grading, as they had been pre-annotated through official pipelines.

In this study, four *FLT3*-related terms are used and defined as follows: (I) *FLT3* mutation refers to the presence of any annotated *FLT3* mutation, including internal tandem duplication (*FLT3*-ITD) and tyrosine kinase domain (*FLT3*-TKD) mutations, and is used for the genomic enrichment and multivariable association analyses; (II) *FLT3*-ITD refers specifically to the clinically annotated *FLT3*-ITD status and is used exclusively in the survival and interaction analyses; (III) *FLT3*-TKD refers specifically to tyrosine kinase domain mutations and was not analyzed as a separate category because of its low frequency; it is captured within the aggregate *FLT3* mutation category; and (IV) *FLT3* alteration is used only as an umbrella term when referring collectively to different types of *FLT3* genomic events and is not used interchangeably with “*FLT3* mutation.”

### 2.4. Pairwise Co-Mutation and Mutual Exclusivity Analysis

To systematically characterize genetic interactions among recurrently mutated genes and explore IF-associated genomic features, pairwise co-occurrence and mutual exclusivity analyses were performed separately within IF and non-IF subgroups. Fisher’s exact test with Benjamini–Hochberg false discovery rate correction was applied to identify significant gene–gene relationships. Only Level 1 and Level 2 pathogenic variants from the CHCMU cohort were included to minimize false-positive signals, while Level 3 variants were excluded. Cross-cohort comparisons were conducted to assess the reproducibility of core genetic interaction signatures between the discovery and validation cohorts.

### 2.5. Gene Fusion Detection and Circos Visualization

Gene fusion events were stratified by IF status in both cohorts. Chromosomal rearrangement patterns and fusion frequency distributions were visualized using Circos plots. Owing to the rarity and high heterogeneity of individual fusion events, fusion alterations were excluded from regression models to prevent sparse-data bias and model overfitting, especially within the limited validation cohort.

### 2.6. Identification of Clinical and Genomic Factors Associated with IF

Univariate and multivariable binary logistic regression models were constructed to identify independent clinical and genomic predictors associated with IF. Candidate predictors included baseline clinical characteristics and recurrent somatic mutations.

Variables with *p* < 0.10 in univariate analyses were considered for multivariable modeling. Patients with missing values in any model covariate were excluded by complete-case deletion, yielding an analytic sample of 617 patients; the final model included sex, WBC > 50 × 10^9^/L, BM blasts > 70%, and *FLT3*, *WT1*, *NPM1*, and *KIT* mutations. To assess the stability of variable selection and address the concern that univariate screening might exclude clinically important covariates, LASSO logistic regression with 10-fold cross-validation and bootstrap resampling (1000 iterations) were performed, and an additional sensitivity analysis forced age, sex, and WBC count into the model together with the genomic candidates (*FLT3*, *WT1*, *NPM1*, and *KIT*), regardless of univariate *p* values. To assess whether cytogenetic risk confounded the mutation–IF association, cytogenetic categories (favorable karyotype [t(8;21)/inv(16)], monosomy 7, normal karyotype) were additionally included in a sensitivity analysis, and cytogenetic aberrations were compared between IF and non-IF patients using Fisher’s exact tests. These analyses were considered exploratory, and *p* values were not adjusted for multiple comparisons.

### 2.7. Survival Analysis

OS between IF and non-IF patients was compared using Kaplan–Meier curves and log-rank tests. Univariable HRs from both cohorts were pooled using a fixed-effects meta-analysis. Multivariable Cox regression in the TARGET cohort was adjusted for age, sex, pretreatment WBC count, and FAB subtype. The proportional hazards assumption was assessed using Schoenfeld residuals. When the assumption was violated, time-varying coefficient or piecewise Cox models were used as appropriate.

Treatment-arm-specific Kaplan–Meier analyses were performed for AAML1031 Arms A (ADE), B (ADE plus bortezomib), and C (ADE plus sorafenib) (regimen details in Table S1), and IF × treatment-arm interactions were assessed using Cox regression. To evaluate whether induction response influenced the prognostic effect of *FLT3*-ITD, an *FLT3*-ITD × IF interaction term was included in the TARGET multivariable Cox model, and subgroup-specific HRs were estimated according to IF status. Similarly, to evaluate whether *NPM1* mutation modified the prognostic impact of *FLT3*-ITD, an *NPM1* × *FLT3*-ITD interaction term was included in multivariable Cox models adjusted for age, sex, WBC count, and FAB subtype, with subgroup-specific HRs estimated according to *NPM1* status; IF rates across *NPM1*/*FLT3*-ITD subgroups were compared using Fisher’s exact test.

Sorafenib exposure (Arm C vs. Arms A/B) was assessed for association with OS using Cox regression, and interactions between sorafenib and IF status or *FLT3*-ITD status were tested. Subgroup analyses were performed among patients with high WBC (>50 × 10^9^/L) and among high-WBC *FLT3*-ITD-positive patients. These analyses were exploratory given the non-randomized nature of Arm C enrollment.

To evaluate the prognostic relevance of HSCT among *FLT3*-ITD-positive patients, EFS and OS were compared between those who received HSCT in first complete remission (SCT-CR1) and those who did not, using landmark analyses at 6 and 12 months to mitigate immortal time bias.

To address potential immortal-time bias arising from the ascertainment of IF at the end of the second induction course, landmark analyses were performed at 60, 90, 120, 150, and 180 days after diagnosis. At each landmark, the risk set was restricted to patients who were alive and at risk, and patients were classified according to their subsequently ascertained IF status at the end of induction course 2. Because the proportional hazards assumption was not satisfied for IF, a piecewise Cox proportional hazards model was additionally fitted, partitioning follow-up into 0–6, 6–24, and >24 months after diagnosis and estimating the IF hazard ratio separately within each interval.

### 2.8. Statistical Analysis

All statistical analyses were performed using R version 4.2.2 and SPSS version 27.0. Continuous variables were compared using appropriate parametric or non-parametric tests, and categorical variables were analyzed using Chi-square or Fisher’s exact tests. Visualization of genomic alterations, interaction networks, and survival analyses was performed using R packages. All statistical tests were two-sided, and *p* < 0.05 was considered statistically significant.

Multiplicity was handled according to a three-tier framework: (I) the Benjamini–Hochberg false discovery rate (FDR) correction was applied to genomic discovery screens involving many simultaneous comparisons—differential mutation frequency, co-mutation/mutual exclusivity, and fusion gene analyses—within each analysis and in both cohorts; (II) a limited set of primary comparisons based on the study aims was not adjusted for multiplicity; and (III) all remaining analyses were considered exploratory, with formal multiplicity adjustment not applied; findings were interpreted based on effect estimates, confidence intervals, internal consistency, cross-validation, and consistency in the independent CHCMU cohort.

## 3. Results

### 3.1. Patient Characteristics

A total of 1979 patients from the TARGET cohort and 98 patients from CHCMU cohort were included in the study. Baseline clinical characteristics of the two cohorts are summarized in Table S2A and Table S2B, respectively.

In the TARGET cohort, patients in the IF group (*n* = 171, 8.6%) were more likely to be male (61.4% vs. 51.3%, *p* = 0.013), younger (median age: 7.8 vs. 10.3 years, *p* = 0.020), and have higher WBC counts (median: 46.8 vs. 25.0 × 10^9^/L, *p* < 0.001) compared with patients in non-IF group (*n* = 1808, 91.4%). Higher BM blasts and peripheral blasts were also observed in IF patients. Notably, a higher proportion of IF patients were classified as higher-risk (29.8% vs. 11.4%, *p* < 0.001). No significant differences were observed in FAB subtype, CNS involvement or treatment protocol between the two groups (all *p* > 0.05).

In the CHCMU cohort, IF patients showed significantly higher BM blasts compared with non-IF patients (median: 60.5% vs. 44.0%, *p* = 0.004). Although not statistically significant due to the limited sample size, trends toward higher male proportion (66.7% vs. 51.9%) and higher risk stratification (52.4% vs. 46.8%) were observed in IF patients, consistent with the TARGET cohort findings. Age, WBC count, FAB subtype, and other clinical characteristics were comparable between the two groups (all *p* > 0.05).

### 3.2. IF Is Associated with Worse OS

**Figure 2:**
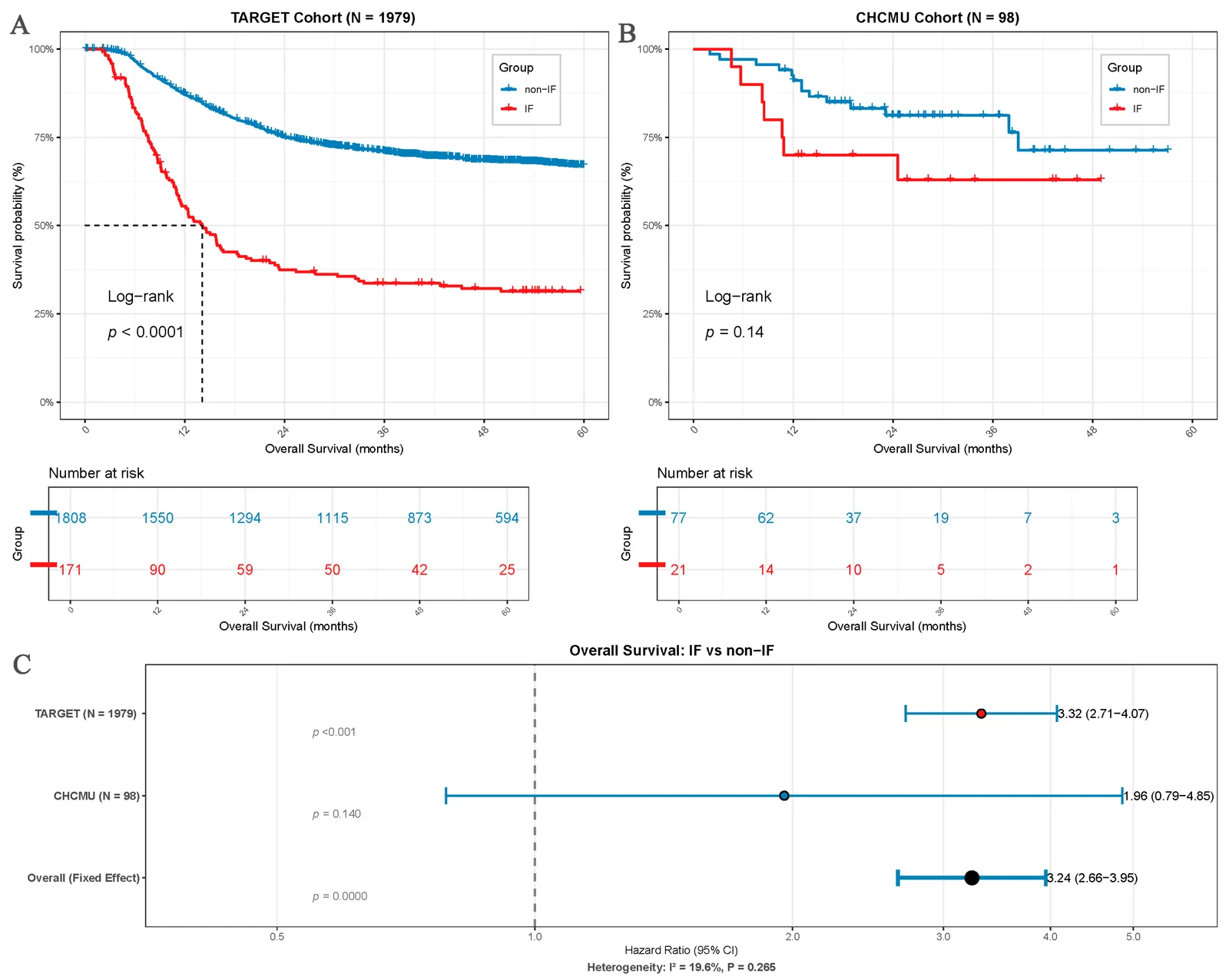
IF is associated with inferior OS in pAML. (**A**) Kaplan–Meier survival curves comparing OS between IF and non-IF patients in the TARGET cohort. (**B**) Kaplan–Meier survival curves comparing OS between IF and non-IF patients in the CHCMU cohort. (**C**) Forest plot showing the meta-analysis of the association between IF status and OS across the two cohorts. The dashed vertical line indicates an HR of 1 (no effect). HRs > 1 indicate increased risk; HRs < 1 indicate reduced risk. Error bars represent 95% confidence intervals.

Given the distinct clinical profiles between IF and non-IF patients, we next evaluated the impact of IF status on OS. As shown in Figure 2A, IF patients in the TARGET cohort had significantly worse OS compared with non-IF patients, with median OS of 12.5 months vs. 45.5 months (HR = 3.32, 95% CI: 2.71–4.07, *p* < 0.001). A consistent trend was observed in the CHCMU cohort (Figure 2B; HR = 1.96, 95% CI 0.79–4.85, *p* = 0.14). Meta-analysis confirmed a consistent association (pooled HR = 3.24, 95% CI: 2.66–3.95, *p* < 0.001), with low heterogeneity (I^2^ = 19.6%, *p* = 0.265; Figure 2C). Collectively, these findings support an adverse prognostic association between IF and OS in pAML.

To address potential immortal-time bias, landmark analyses were performed at 60, 90, 120, 150, and 180 days after diagnosis. In the TARGET cohort, IF remained associated with significantly inferior OS at every landmark (HR = 3.33, 3.23, 3.02, 3.05, and 2.92, respectively; all *p* < 0.001). In the CHCMU cohort, the association was directionally consistent at each landmark (HR range, 1.69–2.32) but did not reach statistical significance, likely reflecting the limited number of IF events (Figure S1).

As the proportional hazards assumption was not satisfied for IF status, a supplementary piecewise Cox analysis with period-specific IF effects (0–6, 6–24, and >24 months after diagnosis) was performed to characterize the temporal pattern of IF-associated mortality risk. IF was associated with the highest mortality risk during the first 6 months after diagnosis (HR = 5.57, 95% CI: 3.58–8.66, *p* < 0.001). The magnitude of risk then decreased over subsequent follow-up periods (6–24 months: HR = 3.29, 95% CI: 2.57–4.23, *p* < 0.001; >24 months: HR = 1.59, 95% CI: 0.81–3.12, *p* = 0.182) (Table S3).

### 3.3. Prognostic Impact of IF Across Treatment Arms

The adverse prognostic association of IF with OS was observed within each treatment arm. However, neither the IF × Arm B interaction nor the IF × Arm C interaction was statistically significant. Within each AAML1031 treatment arm, patients with IF had significantly inferior OS compared with those without IF: ADE alone (Arm A, *p* < 0.0001), ADE plus bortezomib (Arm B, *p* < 0.0001), and ADE plus sorafenib (Arm C, *p* = 0.0052; Figure S2A–C, regimens and arm sizes in Table S1). In Cox regression models including IF × treatment-arm interaction terms, neither the IF × Arm B interaction (HR = 1.58, 95% CI, 0.84–2.99; *p* = 0.157) nor the IF × Arm C interaction (HR = 1.28, 95% CI, 0.43–3.77; *p* = 0.66) was statistically significant (Figure S2D).

To assess whether sorafenib exposure modified the IF–OS association, we compared Arm C (ADE + sorafenib) with Arms A/B. Sorafenib exposure was not associated with a significant OS difference overall (HR = 1.17, 95% CI 0.77–1.77, *p* = 0.47), and the IF × Arm C interaction was not statistically significant (*p* = 0.96). Because Arm C enrolled only patients with HAR *FLT3*-ITD, a formal sorafenib × *FLT3*-ITD interaction was estimated with limited precision and was not statistically significant (*p* = 0.45); OS by sorafenib exposure was therefore additionally examined within *FLT3*-ITD-positive and high-WBC subgroups (Figure S3A–D). Figure S3A shows the comparison among all *FLT3*-ITD-positive patients, in whom sorafenib exposure was not associated with OS (HR = 0.97, 95% CI 0.46–2.08, *p* = 0.944). Figure S3B shows the comparison among all high-WBC patients (HR = 1.38, 95% CI 0.83–2.28, *p* = 0.212). Figure S3C shows the comparison among the 55 high-WBC *FLT3*-ITD-positive patients (15 vs. 40), in whom the IF rate was numerically lower in sorafenib-exposed patients (0/15 [0%] vs. 4/40 [10.0%]; Fisher’s exact test *p* = 0.57), with no significant OS difference (HR = 0.78, 95% CI 0.29–2.15, *p* = 0.637). Figure S3D shows the comparison among *FLT3*-ITD-positive patients with low WBC, in whom no significant difference was observed (*p* = 0.980). These analyses were underpowered and should be considered exploratory.

### 3.4. Mutational Landscape of IF and Non-IF Patients

**Figure 3:**
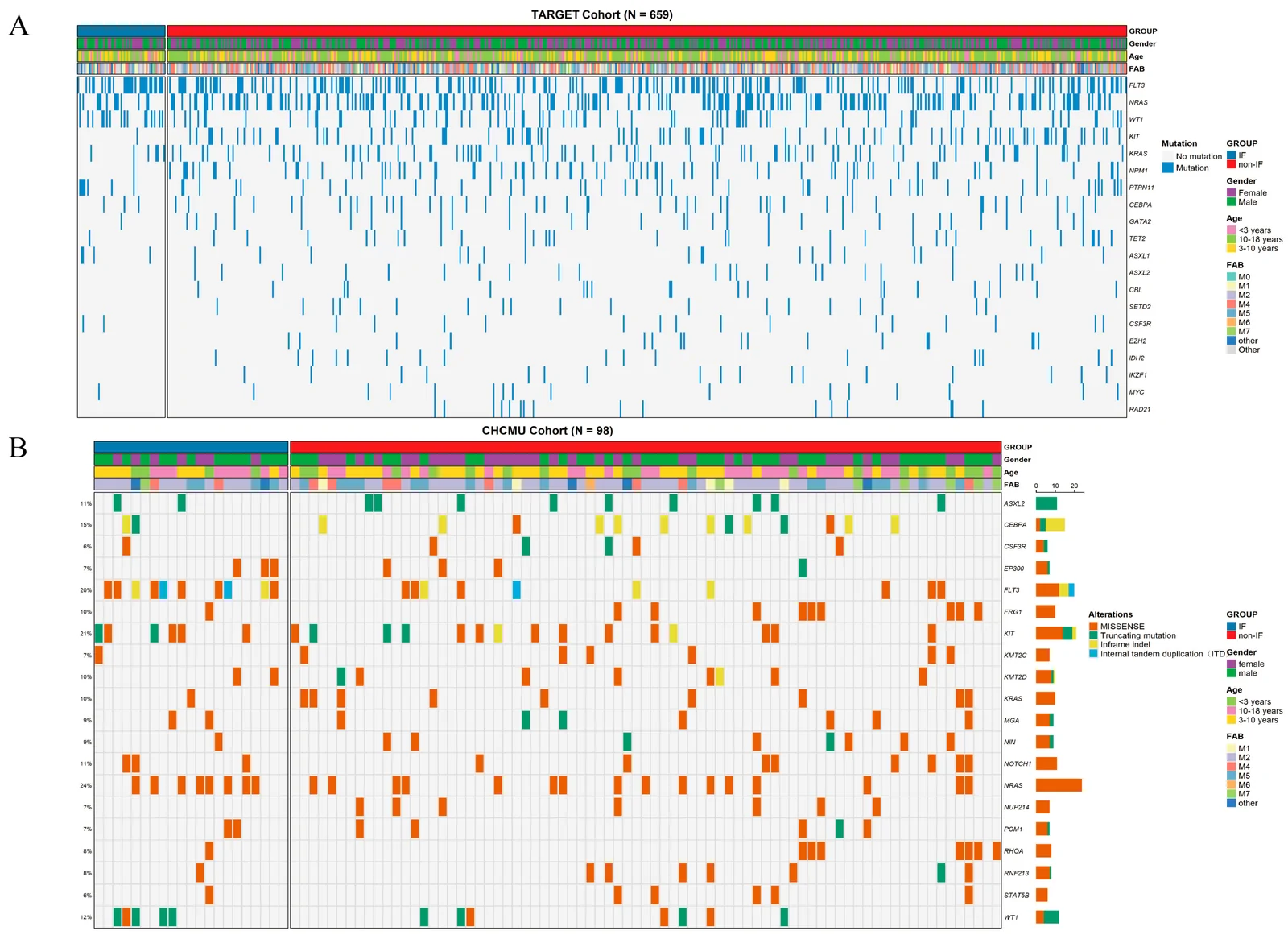
Genomic landscape of pAML in the TARGET and CHCMU cohorts. (**A**) Oncoplot showing recurrent somatic mutations in the TARGET cohort. (**B**) Distribution of pathogenic and likely pathogenic mutations identified in the CHCMU cohort.

We first characterized the overall genomic mutational profiles of pAML patients using targeted capture sequencing data for the TARGET cohort and RNA-seq data for the CHCMU cohort. In the TARGET cohort, the top five most frequently mutated genes were *FLT3* (33.1%), *NRAS* (30.3%), *WT1* (14.0%), *KIT* (13.2%), and *KRAS* (12.4%) (Figure 3A). For the CHCMU validation cohort, a total of 1101 somatic mutation events across 462 genes were identified and stratified into three pathogenicity levels: 125 (11.4%) level 1 (clearly pathogenic), 86 (7.8%) level 2, and 890 (80.8%) level 3. Detailed clinical features of level 1 and level 2 mutations are summarized in Table S4. Among level 1 mutations, six genes were recurrently mutated in over 10% of patients: *NRAS* (23.5%), *KIT* (19.4%), *FLT3* (17.3%), *WT1* (11.2%), *CEBPA* (10.2%), and *KRAS* (10.2%). Notably, in the CHCMU cohort, among patients with *FLT3* mutations (level 1), 58.8% (10/17) were in the IF group and 41.2% (7/17) in the non-IF group; conversely, among patients with *CEBPA* mutations (level 1), 90.0% (9/10) were in the non-IF group and 10.0% (1/10) in the IF group. *KIT* mutations showed a strong correlation with FAB-M2 subtype, with 84.2% of *KIT*-mutated cases classified as M2. For level 2 variants, the most prevalent genes were *ASXL2* (11.2%), *FLT3* (5.1%), *CSF3R* (4.1%), *CEBPA* (4.1%), and *GATA2* (3.1%). Level 1 mutations thus serve as the core drivers, while additional level 2 alterations may cooperate in disease progression (Figure 3B, Table S4).

**Figure 4:**
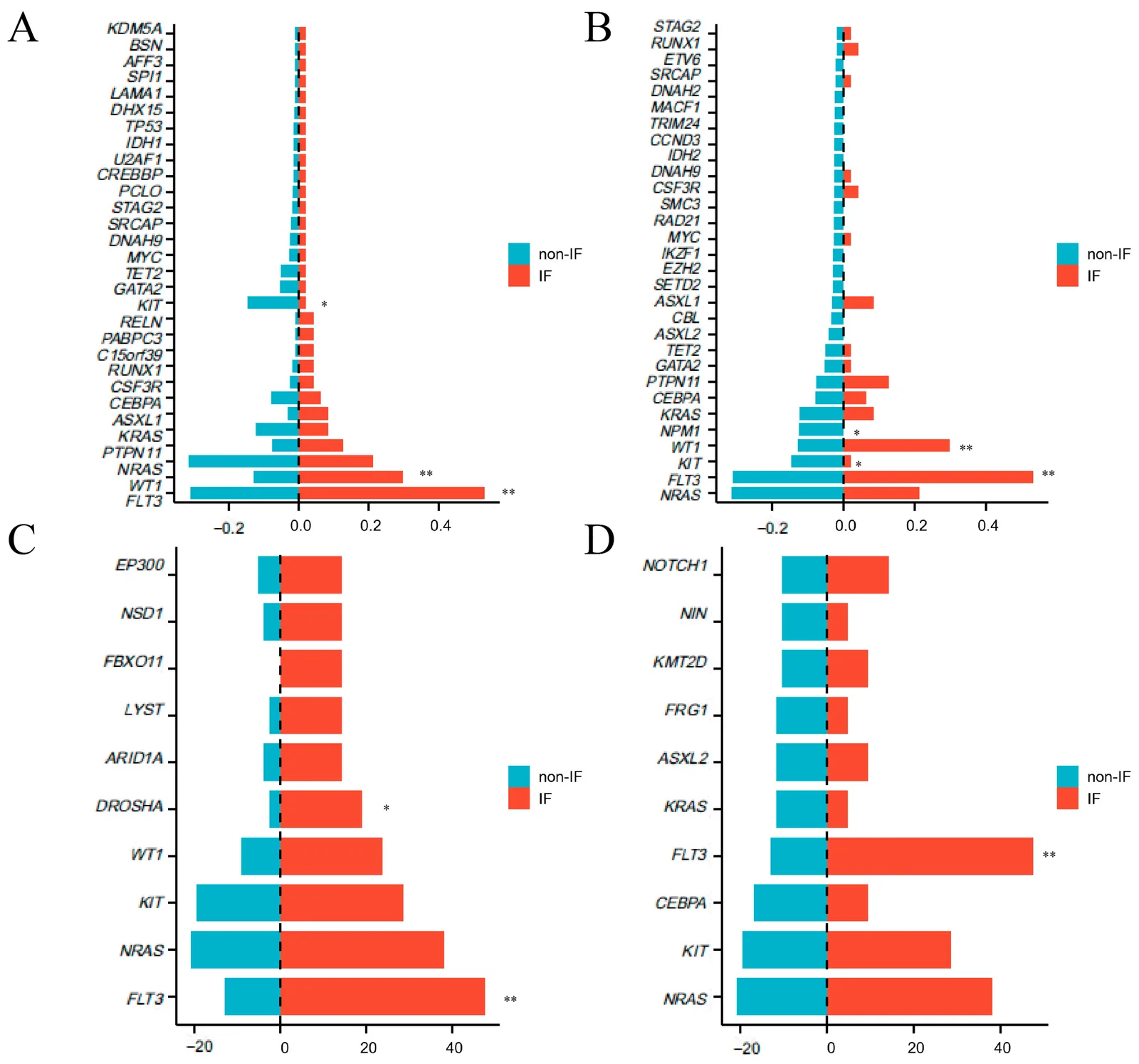
Differential mutation profiles between IF and non-IF patients. Comparison of recurrent somatic mutation frequencies between IF and non-IF patients in the TARGET discovery cohort (**A**,**B**) and CHCMU validation cohort (**C**,**D**). Statistical significance was assessed using Fisher’s exact test with Benjamini–Hochberg correction. Asterisks indicate statistical significance: * *p* < 0.05, ** *p* < 0.01.

We next compared mutational profiles between IF and non-IF patients in the TARGET cohort. Among 55 IF and 604 non-IF patients, the top 30 most frequently mutated genes ranked by IF group frequency are shown in Figure 4A. *FLT3* and *WT1* mutations were significantly more frequent, whereas *KIT* mutations were significantly less frequent, in the IF group than in the non-IF group (all *p* < 0.05). These patterns remained consistent when genes were ranked by non-IF group frequency (Figure 4B).

The same analytical strategy was applied to the CHCMU cohort. Due to the limited sample size, we focused on the top 10 most frequently mutated genes ranked by IF group frequency. IF patients showed significantly higher frequencies of *FLT3* and *DROSHA* mutations compared with non-IF patients (Figure 4C). The enrichment of *FLT3* mutations in the IF group was further validated when genes were ranked by non-IF group frequency (Figure 4D), further supporting the association between *FLT3* mutation and IF in pAML.

### 3.5. Distinct Genomic Interaction Patterns Associated with IF

To characterize genomic interaction patterns associated with induction response, pairwise co-mutation and mutual exclusivity analyses were performed in both cohorts. For the CHCMU cohort, only Level 1 and Level 2 pathogenic variants were included to minimize false-positive signals.

In the TARGET cohort, patients were stratified into non-IF (*n* = 604) and IF (*n* = 55) subgroups. Among non-IF patients, multiple recurrent interaction patterns were identified. After FDR correction, ten gene pairs remained significant (Figure S4; Table S5), including six co-occurring pairs (*CEBPA*–*GATA2*, *FLT3*–*NPM1*, *CEBPA*–*CSF3R*, *FLT3*–*WT1*, *NPM1*–*IDH2*, and *NPM1*–*PTPN11*) and four mutually exclusive pairs (*FLT3*–*KIT*, *KIT*–*NPM1*, *FLT3*–*KRAS*, and *FLT3*–*NRAS)*.

In contrast, the IF subgroup showed a substantially reduced interaction landscape, with no gene pairs remaining significant after multiple testing correction. This pattern may reflect distinct genomic characteristics of chemo-resistant disease, although the limited number of IF cases may have reduced statistical power.

Consistent with these findings, *FLT3* and *WT1* mutations were enriched in IF patients, whereas *KIT* and *NPM1* mutations were more frequently observed in non-IF patients (Figure 4). In the independent CHCMU cohort, although no interaction remained significant after FDR correction, *FLT3*–*WT1* co-occurrence and *CEBPA*–*KIT* mutual exclusivity showed consistent directional trends with the TARGET cohort (Figure S5; Table S5). These findings provide exploratory evidence of broadly consistent *FLT3*-centered genomic interaction patterns across cohorts.

### 3.6. Fusion Gene Distribution Associated with Induction Response

**Figure 5:**
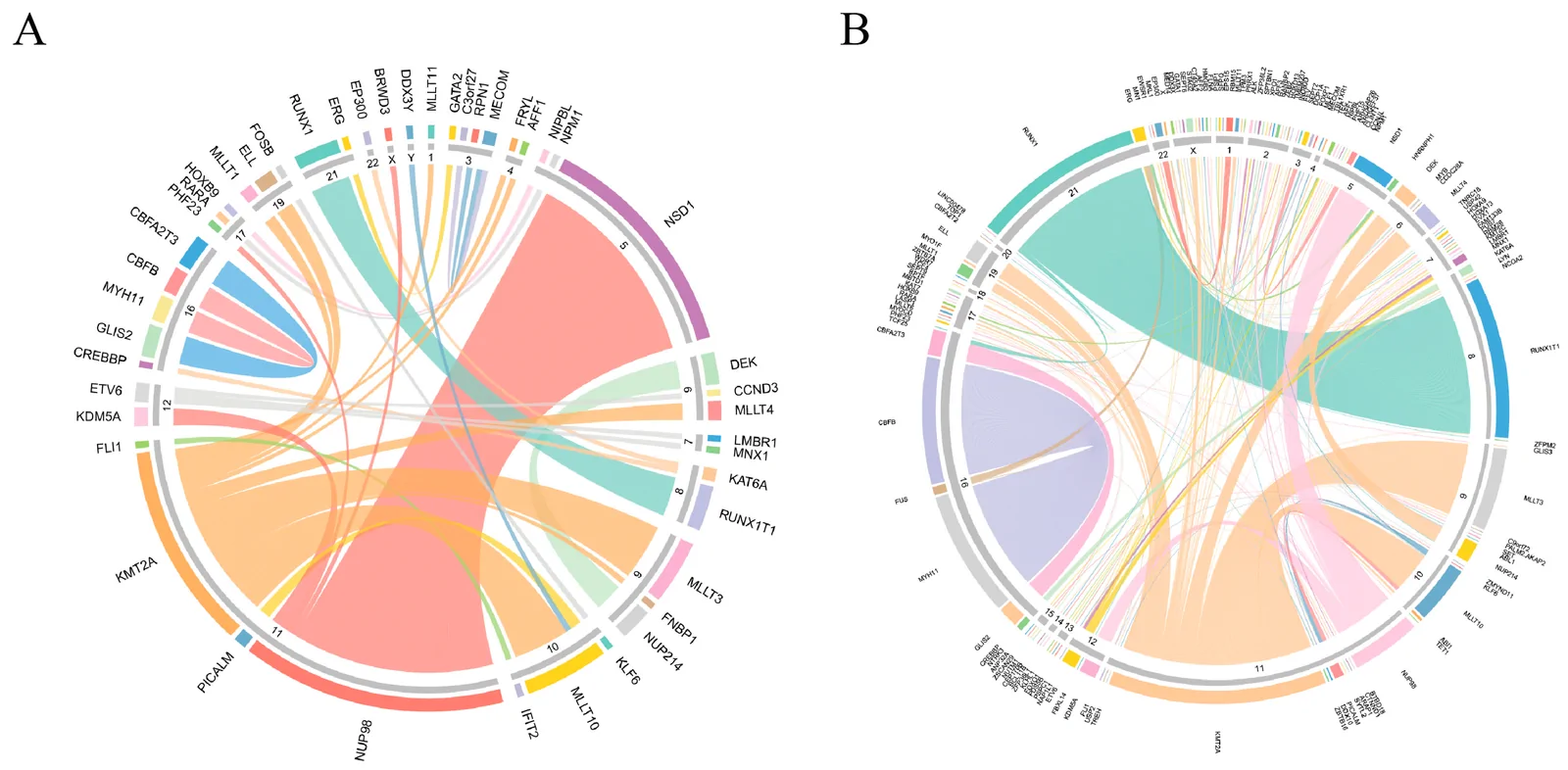
Fusion gene landscapes associated with IF. Circos plots illustrating chromosomal rearrangements and fusion gene distributions in IF (**A**) and non-IF (**B**) patients from the TARGET discovery cohort. Colors indicate different chromosomes; grey sectors group genes by chromosome. Ribbons represent fusion events, and arrows indicate the direction of the fusion (from the 5′ partner gene to the 3′ partner gene).

Given the important role of gene fusions in pAML, we compared fusion landscapes between IF and non-IF patients in both cohorts. Fusion patterns were visualized using Circos plots (TARGET: Figure 5A,B; CHCMU: Figure S6A,B). Differing from the targeted mutation analysis, fusion genes detected in the CHCMU cohort were further stratified into level 1, level 2, and level 3 variants based on authoritative AMP/ASCO/CAP criteria and Chinese expert consensus (Table S6), while no variant-level classification was performed for fusion data in the TARGET cohort.

In the TARGET cohort, distinct fusion profiles were observed between IF and non-IF patients. IF patients showed enrichment of adverse-risk fusions, particularly *NUP98*-*NSD1* (OR = 8.38, 95% CI: 5.07–13.74, *p* < 0.001), whereas non-IF patients were characterized by favorable-risk rearrangements, including *RUNX1*-*RUNX1T1* (OR = 0.23, 95% CI: 0.09–0.50, *p* < 0.001) and *CBFB*-*MYH11* (OR = 0.18, 95% CI: 0.05–0.47, *p* < 0.001). These fusion events showed significant associations with induction response after multiple testing correction (Table S7).

In the CHCMU validation cohort, RNA-seq identified 124 fusion events involving 80 genes. Although recurrent fusion patterns differed from the TARGET cohort, the overall direction of association was difficult to evaluate due to the limited number of IF cases and the low frequency of individual fusion events. No recurrent fusion reached statistical significance after FDR correction.

### 3.7. Factors Associated with IF

To identify independent predictors of IF in pAML, we conducted univariate and multivariable logistic regression in the TARGET discovery and CHCMU validation cohorts, incorporating only clinical variables and somatic gene mutations as candidate predictors. All factors with univariate *p* < 0.1 entered multivariate models.

In the TARGET cohort, univariate analysis showed that *FLT3* and *WT1* mutations, WBC > 50 × 10^9^/L, and higher BM blast percentage increased IF risk, while *NPM1*, *KIT* mutations and female sex reduced IF risk. Multivariable regression identified *FLT3* (OR = 3.18, *p* < 0.001), *WT1* (OR = 2.11, *p* = 0.042) mutations and female sex (OR = 0.47, *p* = 0.024) as independent IF predictors (Figure S7; Table 1).

In the CHCMU validation cohort, only *FLT3* mutation was associated with IF in univariate analysis (OR = 6.09, *p* = 0.001), with *WT1* mutation showing a marginal trend (*p* = 0.079). Due to limited IF patients (*n* = 21), only these two mutated genes were included in multivariable regression to avoid overfitting. *FLT3* mutation remained an independent risk factor (OR = 5.361, *p* = 0.003), whereas the trend of *WT1* mutation vanished after adjustment (*p* = 0.382). No other clinical or mutational features correlated with IF in the validation cohort (Figure S8; Table 1).

Across the two cohorts, *FLT3* mutation showed a consistent independent association with IF. In contrast, the associations of *WT1* mutation and female sex with IF were observed in the TARGET cohort but were not reproduced in the CHCMU cohort, potentially reflecting limited statistical power. Collectively, these findings identify *FLT3* mutation as the most consistent genomic correlate of IF across the two cohorts.

To assess the stability of variable selection, LASSO logistic regression with 10-fold cross-validation and bootstrap resampling (1000 iterations) was performed. *FLT3* was re-selected in 99% of bootstrap resamples, and *WT1* at a high frequency (91%), consistent with the primary multivariable model. WBC >50 × 10^9^/L was also among the most frequently selected variables (96%; Figure S9).

In the forced-entry model (age, sex, WBC count, *FLT3*, *WT1*, *NPM1*, and *KIT* mutation) fitted in the same analytic sample as the primary multivariable model, the estimates for *FLT3* (OR = 3.10, 95% CI 1.56–6.16, *p* = 0.001) and *WT1* (OR = 2.03, 95% CI 0.98–4.20, *p* = 0.058) were essentially unchanged. These multivariable regression analyses were performed as association analyses rather than predictive models; accordingly, the results should be interpreted with respect to the direction, magnitude, and statistical significance of the associations.

### 3.8. Cytogenetic Factors Associated with IF

To assess whether the higher IF rate in the CHCMU cohort reflected enrichment of unfavorable cytogenetic risk, we compared IF rates by cytogenetic category between cohorts using Fisher’s exact tests. IF rates were significantly higher in the CHCMU cohort among favorable-risk t(8;21) (16.0% vs. 3.0%, *p* = 0.028), inv(16) (33.3% vs. 1.0%, *p* = 0.008), and normal-karyotype (30.0% vs. 12.3%, *p* = 0.023) subgroups, and numerically higher for *KMT2A*-rearranged cases (30.0% vs. 7.7%, *p* = 0.053), whereas rates were similar for other cytogenetic abnormalities (11.1% vs. 12.2%, *p* > 0.99) (Figure S10). *p* values were not adjusted for multiple comparisons and should be considered exploratory.

Among 659 TARGET patients with available karyotypes, favorable-risk core-binding factor karyotypes were significantly less frequent in IF patients (t(8;21): 5.5% vs. 16.2%, OR = 0.34, *p* = 0.031; inv(16): 1.8% vs. 15.9%, OR = 0.11, *p* = 0.002), whereas monosomy 7 (9.1% vs. 1.7%, OR = 5.49, *p* = 0.005) and normal karyotype (36.4% vs. 23.7%, OR = 1.54, *p* = 0.049) were more frequent (Table S8).

In multivariable logistic regression adjusted for age, sex, and WBC count, inv(16) (OR = 0.09, 95%CI 0.01–0.69, *p* = 0.02) and monosomy 7 (OR = 6.13, 95% CI 1.78–21.05, *p* = 0.004) remained independent predictors of IF, whereas t(8;21) did not reach significance after adjustment (OR = 0.38, *p* = 0.14) (Table S9).

To directly test whether cytogenetics confound the *FLT3*–IF association, we extended the primary multivariable model (Table 1) by additionally including cytogenetic categories (favorable karyotype, monosomy 7, normal karyotype). *FLT3* mutation remained independently associated with IF after this adjustment (OR = 2.50, 95% CI 1.21–5.15, *p* = 0.013), supporting the persistence of the *FLT3*–IF association after adjustment for major cytogenetic categories (Table S10).

### 3.9. Prognostic Significance of FLT3-ITD According to Induction Response and NPM1 Status

**Figure 6:**
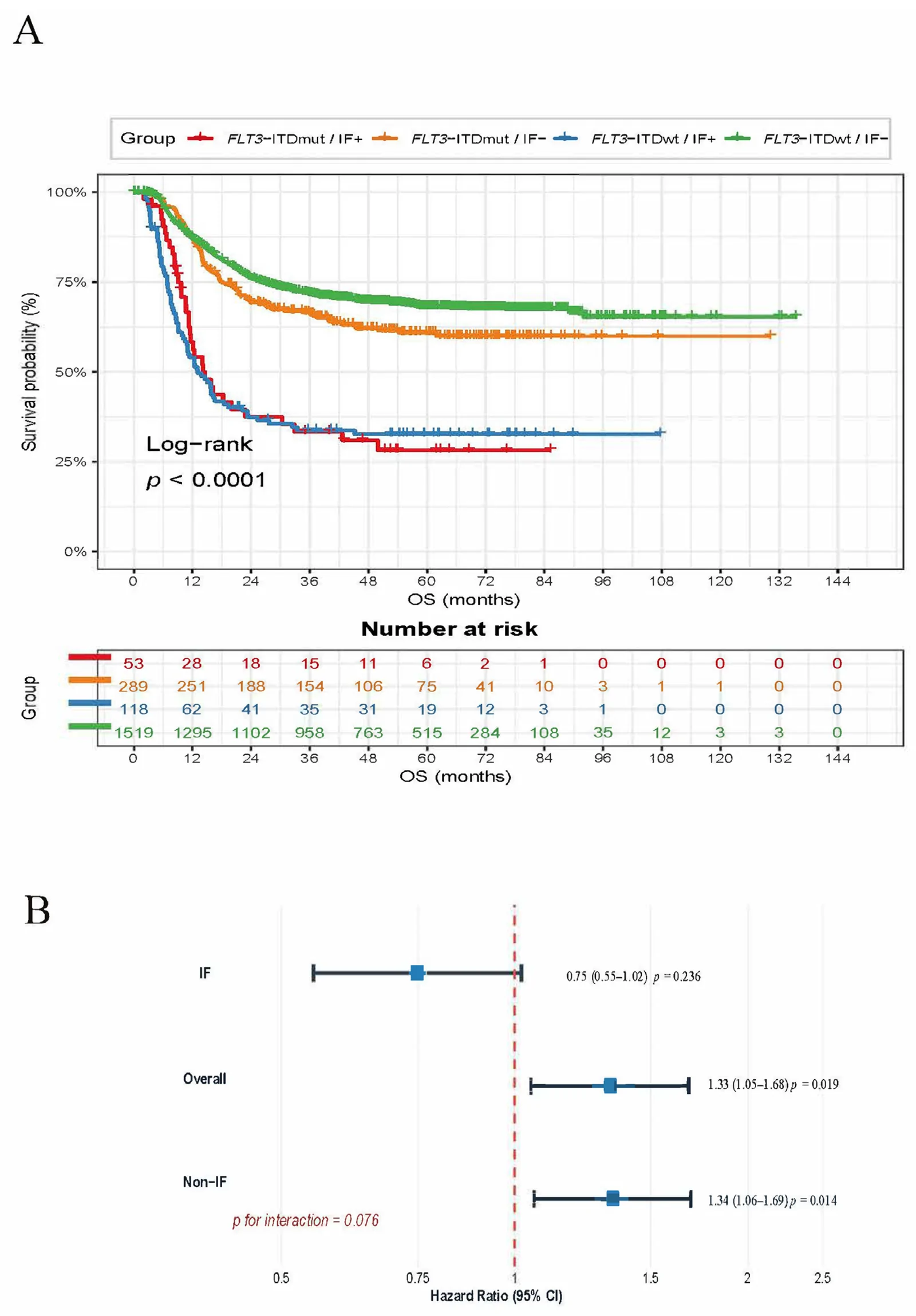
Prognostic significance of *FLT3*-ITD according to induction response in pAML. (**A**) Kaplan–Meier curves showing OS according to *FLT3*-ITD and IF status. Patients were stratified into four groups: *FLT3*-ITD+/IF+, *FLT3*-ITD+/IF−, *FLT3*-ITD−/IF+, and *FLT3*-ITD−/IF−. (**B**) Forest plot showing the prognostic effect of *FLT3*-ITD on OS stratified by IF status. Hazard ratios (HRs) and 95% confidence intervals (CIs) were estimated using multivariable Cox regression models adjusted for age, sex, white blood cell count, and FAB subtype. *p* for interaction between *FLT3*-ITD status and IF status is shown.

To investigate whether the prognostic relevance of *FLT3*-ITD differed according to induction response status, patients were stratified into four subgroups based on *FLT3*-ITD status and IF status: *FLT3*-ITD+/IF+, *FLT3*-ITD+/IF−, *FLT3*-ITD−/IF+, and *FLT3*-ITD−/IF−. Kaplan–Meier survival analysis demonstrated a significant difference in OS among the four groups (Figure 6A). Within the IF subgroup, *FLT3*-ITD status was not associated with significant OS differences (*p* = 0.78; Figure S11A). In contrast, among non-IF patients, *FLT3*-ITD-positive cases showed significantly inferior OS compared with *FLT3*-ITD-negative cases (*p* = 0.02; Figure S11B).

To further evaluate whether IF status influenced the prognostic association of *FLT3*-ITD, multivariable Cox regression models adjusted for age, sex, WBC count, and FAB subtype were performed. Among non-IF patients, *FLT3*-ITD was independently associated with inferior OS (HR = 1.34, 95% CI: 1.06–1.69, *p* = 0.014). Conversely, among patients with IF, *FLT3*-ITD did not confer additional survival risk (HR = 0.75, 95% CI: 0.55–1.02, *p* = 0.236) (Figure 6B). The interaction between *FLT3*-ITD and IF status was not statistically significant (*p* for interaction = 0.076), although *FLT3*-ITD remained independently associated with inferior OS among non-IF patients.

Given the reported clinical interplay between *NPM1* mutation and *FLT3*-ITD in AML, we evaluated whether *NPM1* mutation modified the prognostic impact of *FLT3*-ITD. In the TARGET cohort, *FLT3*-ITD was associated with inferior OS in *NPM1*-wildtype patients (HR = 1.50, 95% CI: 1.24–1.82, *p* < 0.001), whereas the association was not statistically significant in *NPM1*-mutated patients (HR = 1.20, 95% CI: 0.56–2.55, *p* = 0.64). The *NPM1* × *FLT3*-ITD interaction term was not statistically significant (HR = 0.80, 95% CI: 0.36–1.74, *p* = 0.566) (Table S11A; Figure S12A). Cox models adjusted for age, sex, WBC count, and FAB subtype yielded consistent results (interaction *p* = 0.53).

We further evaluated IF rates across the four *NPM1*/*FLT3*-ITD subgroups among the TARGET patients with validated *NPM1* genotypes: WT/ITD−, 6.3% (31/491); WT/ITD+, 24.7% (23/93); Mut/ITD−, 2.3% (1/44); and Mut/ITD+, 0.0% (0/30). Among *NPM1*-wildtype patients, *FLT3*-ITD was associated with a higher IF rate (24.7% vs. 6.3%). Among *NPM1*-mutated patients, no increase in IF rate was observed in the *FLT3*-ITD-positive subgroup (0.0% vs. 2.3%), although the number of *NPM1*-mutated patients with *FLT3*-ITD was small. The IF rate among *FLT3*-ITD-positive patients differed significantly according to *NPM1* status (24.7% [23/93] vs. 0.0% [0/30]; Fisher’s exact test, *p* = 0.001) (Table S11B; Figure S12B). Given the limited number of *NPM1*-mutated patients, particularly those with *FLT3*-ITD, these subgroup findings should be interpreted cautiously.

### 3.10. Association of HSCT with Outcomes in FLT3-ITD-Positive Patients Without IF

Given the persistent adverse prognostic significance of *FLT3*-ITD among patients achieving induction remission, we further evaluated whether HSCT was associated with outcomes in *FLT3*-ITD-positive patients. This analysis was restricted to *FLT3*-ITD-positive patients without IF who had documented CR1 transplant status. Among 275 such patients (14 with unknown transplant status excluded), 135 underwent HSCT in CR1 and 140 did not. HSCT was associated with significantly improved EFS, with 3-year EFS rates of 62.0% vs. 40.8% in transplanted and non-transplanted patients, respectively (HR = 0.51, 95% CI: 0.36–0.72, *p* < 0.001; Figure S13A). In contrast, OS was not significantly different between the two groups, although a favorable trend was observed in patients who underwent HSCT (3-year OS, 70.9% vs. 63.4%; HR = 0.74, 95% CI: 0.50–1.10, *p* = 0.14; Figure S13B).

To address potential time-related bias associated with transplantation, landmark analyses were performed. At the 6-month landmark, HSCT remained significantly associated with improved EFS (HR = 0.57, *p* = 0.003), whereas the association with OS did not reach statistical significance (HR = 0.69, *p* = 0.081). At the 12-month landmark, neither EFS (HR = 0.66, *p* = 0.117) nor OS (HR = 0.74, *p* = 0.227) differed significantly between the two groups.

## 4. Discussion

In this multi-cohort study, we systematically characterized the clinical and genomic determinants of IF in pAML by integrating a large TARGET discovery cohort with an independent CHCMU validation cohort. Our study demonstrates that IF represents a clinically distinct high-risk phenotype associated with extremely poor survival outcomes. Through comprehensive genomic analyses, we identified distinct molecular characteristics associated with induction response, including enrichment of adverse genetic alterations, differential fusion patterns, and altered gene interaction landscapes. Importantly, *FLT3* mutation emerged as the most consistent genomic correlate of IF across the two independent cohorts. We further examined the prognostic associations of *FLT3*-ITD in the context of induction response, providing insight into the relationship between baseline molecular risk and early treatment response.

IF represents one of the most clinically relevant manifestations of treatment resistance in pAML [25]. In our study, IF patients exhibited substantially inferior OS compared with non-IF patients, and this association was consistently observed in both Western and Chinese cohorts [13,18,19]. Taken together, our data support the concept that IF is not merely an intermediate treatment response parameter but rather reflects a clinically defined resistant disease state. The poor outcomes associated with IF may reflect persistence of chemotherapy-resistant leukemic populations despite intensive induction therapy. Failure to achieve remission may indicate incomplete eradication of malignant populations, resulting in a higher risk of subsequent disease progression and fewer opportunities for effective consolidation strategies [14,21,26].

Interestingly, the time-dependent analysis showed that the prognostic impact of IF was greatest in the early post-diagnosis period and attenuated over time. The weaker association observed at later time points may reflect, at least in part, survivor selection and changes in the composition of the at-risk population over time. Current pAML risk stratification relies largely on baseline clinical and genomic features [27,28,29] and may not fully capture intrinsic resistance to induction therapy; although the importance of early treatment response is increasingly recognized, the molecular determinants of IF remain insufficiently characterized [30]. Our findings indicate that early response provides complementary biological information beyond baseline genomics, supporting integration of dynamic response measures with molecular risk factors in risk-adapted treatment planning.

By comparing genomic profiles between IF and non-IF patients, we identified distinct molecular characteristics associated with induction response. Among recurrently mutated genes, *FLT3* demonstrated the strongest and most consistent association with IF across both the TARGET and CHCMU cohorts. *FLT3* activation promotes leukemic cell proliferation and survival through downstream signaling pathways, including MAPK and PI3K/AKT signaling, which may contribute to reduced chemotherapy sensitivity [31]. *WT1* mutations were also enriched among IF patients in the discovery cohort, consistent with previous studies linking *WT1* abnormalities to adverse biological features [13,25,32]; however, this association was not independently confirmed in the validation cohort, likely reflecting limited statistical power. Cytogenetic analysis confirmed that favorable-risk core-binding factor karyotypes were associated with lower IF risk, whereas monosomy 7 was associated with higher IF risk, consistent with the known prognostic significance of these aberrations in pAML. Cytogenetic sensitivity analysis further showed that the association of *FLT3* mutation with IF remained statistically significant after adjustment for major cytogenetic categories, with the OR decreasing from 3.18 to 2.50 (95% CI 1.21–5.15, *p* = 0.013).

Beyond individual genomic alterations, our study revealed distinct genetic interaction landscapes between IF and non-IF patients. Non-IF patients displayed relatively conserved co-mutation structures, including recurrent associations such as *FLT3*–*NPM1* and *CEBPA*–*GATA2*, together with mutual exclusivity between *FLT3* and *KIT* or RAS pathway genes. These patterns are consistent with previously reported genomic relationships in AML and may reflect the genetic structure of distinct molecular subgroups [33]. In contrast, IF patients showed a relative reduction in recurrent co-mutation and mutual exclusivity patterns. Induction resistance may therefore not be attributable to a single dominant genomic pathway but instead arise across diverse molecular backgrounds [34,35,36]. Although the limited number of IF cases restricted the statistical power of interaction analyses, the distinct interaction landscape observed in IF patients supports the molecular heterogeneity of primary chemotherapy resistance.

Fusion profiling further supported the molecular distinction between IF and non-IF patients. In the TARGET cohort, *NUP98*-*NSD1* rearrangement was enriched among IF patients, whereas favorable-risk core-binding factor (CBF) fusions, including *RUNX1*-*RUNX1T1* and *CBFB*-*MYH11*, were predominantly observed among non-IF patients. This distribution is consistent with previous studies demonstrating that *NUP98*-rearranged AML represents a high-risk molecular subtype associated with inferior treatment response, whereas CBF AML generally exhibits favorable chemotherapy sensitivity [11,37,38,39,40]. Notably, *NUP98*-*NSD1* frequently co-occurs with *FLT3*-ITD, and previous studies have suggested that these alterations may cooperate to promote aggressive disease biology. The enrichment of *NUP98*-*NSD1* among IF patients in our study further supports the contribution of high-risk fusion events to primary chemotherapy resistance [4,41].

However, these fusion associations were not statistically validated in the CHCMU cohort. This discrepancy is likely attributable to the rarity of individual fusion events and the limited number of IF cases in the validation cohort, which substantially reduced statistical power. In addition, differences in genetic background and disease subtype distribution between populations may contribute to variation in fusion frequencies. Therefore, larger international multicenter cohorts will be required to determine whether specific fusion alterations represent universal determinants of induction resistance or population-specific biomarkers. Nevertheless, the enrichment patterns observed in the discovery cohort provide biological insights into the molecular heterogeneity underlying IF.

The higher IF rate observed in the CHCMU cohort compared with the TARGET cohort is likely multifactorial. First, the CHCMU cohort was restricted to patients with available transcriptome data, which requires adequate cellularity and thus may preferentially include patients with higher disease burden—a known risk factor for IF. Second, only patients who completed two induction courses were evaluable; the exclusion of early deaths and early withdrawals may have altered the composition of the evaluable population relative to the TARGET cohort. Third, as a single-institution real-world cohort, CHCMU may contain a higher proportion of high-risk, treatment-refractory, or referral patients, and local practice regarding the timing and delivery of the second induction course may differ from the standardized TARGET protocols. The same IF definition was applied in both cohorts, supporting the internal validity of the IF–OS comparison; nevertheless, the absolute IF rate and its cross-cohort differences should be interpreted with caution given their dependence on these cohort-specific factors.

In the CHCMU cohort, salvage chemotherapy after IF consisted primarily of cladribine-based regimens (C-HAG) in 16 of 21 patients (76.2%), and 6 of 21 patients (28.6%) subsequently proceeded to HSCT. Salvage treatment exposure was not significantly associated with any of the key mutated genes examined (*FLT3*, *WT1*, *KIT*, *NRAS*, and *CEBPA*; all *p* > 0.05, Table S12), providing no evidence for a genotype-by-salvage interaction. However, because the salvage regimens were relatively uniform and the number of IF patients was small (*n* = 21), formal genotype-by-treatment interaction modeling was not feasible, and these descriptive comparisons should be regarded as supportive rather than definitive.

A major finding of this study was that *FLT3*-ITD remained associated with inferior survival among patients who achieved induction remission. *FLT3*-ITD has traditionally been considered an adverse molecular alteration associated with increased relapse risk and inferior survival in AML [42]. However, previous studies have generally evaluated *FLT3*-ITD as a baseline molecular marker without explicitly considering early treatment response. In our analysis, *FLT3*-ITD was associated with significantly inferior survival among patients who achieved induction remission, indicating that its adverse prognostic association persisted despite successful initial treatment. In contrast, no statistically significant additional prognostic association of *FLT3*-ITD was detected among patients with IF. Importantly, the formal *FLT3*-ITD × IF interaction did not reach statistical significance (*p* = 0.076); therefore, these findings do not establish that the prognostic association of *FLT3*-ITD differs according to induction-response status. One possible explanation is that the strong adverse prognosis associated with failure to achieve remission may limit the additional prognostic discrimination attributable to *FLT3*-ITD, although this interpretation remains hypothesis-generating.

Consistent with the literature and with ELN risk-stratification principles [43], the observed association between *FLT3*-ITD and IF varied descriptively across *NPM1* subgroups. Among *NPM1*-wildtype patients, the IF rate was higher in those with *FLT3*-ITD than in those without *FLT3*-ITD (24.7% vs. 6.3%). In contrast, among *NPM1*-mutated patients, no increase in IF rate was observed in the *FLT3*-ITD-positive subgroup (0.0% vs. 2.3%), although this subgroup included only 30 patients. The IF rate among *FLT3*-ITD-positive patients differed significantly according to *NPM1* status (24.7% [23/93] vs. 0.0% [0/30]; Fisher’s exact test, *p* = 0.001). However, the formal *NPM1* × *FLT3*-ITD interaction was not statistically significant for OS (*p* = 0.566; adjusted *p* = 0.53). These findings therefore remain exploratory and should be interpreted cautiously, particularly given the small number of *NPM1*-mutated patients and the absence of IF events among *NPM1*-mutated/*FLT3*-ITD-positive patients. Taken together, these findings underscore the importance of considering baseline molecular features together with early treatment response when interpreting prognostic risk in pAML [43].

These findings may have implications for molecularly informed risk stratification and therapeutic decision-making in pAML. *FLT3*-directed therapies have demonstrated clinical activity in *FLT3*-ITD-positive pAML; notably, the COG AAML1031 trial reported improved outcomes with the addition of sorafenib to conventional chemotherapy in children with high-allelic-ratio *FLT3*-ITD-positive AML [44]. However, given the high rate of transplantation in AAML1031, the extent to which the observed benefit can be attributed specifically to *FLT3* inhibition remains difficult to disentangle. Our study did not evaluate *FLT3*-targeted therapy or treatment-specific efficacy and therefore cannot determine whether *FLT3* inhibition modifies the prognostic risk identified in our analysis. Importantly, the adverse prognostic association of IF was consistent across the AAML1031 treatment arms, including the sorafenib-containing arm, with no significant IF × treatment-arm interaction (IF × Arm B: HR = 1.58, 95% CI 0.84–2.99, *p* = 0.157; IF × Arm C: HR = 1.28, 95% CI 0.43–3.77, *p* = 0.66; Figure S2D). Among patients who achieve induction remission, *FLT3*-ITD identifies a subgroup with persistent adverse prognostic association, highlighting the potential relevance of integrating treatment response with molecular risk assessment. In contrast, among patients with IF, the profound adverse prognosis associated with induction resistance may overshadow the additional prognostic contribution of *FLT3*-ITD, suggesting that mechanisms beyond *FLT3* signaling may also contribute to treatment resistance [45,46]. More broadly, these findings support a dynamic approach to molecular risk assessment in which baseline genomic features are interpreted together with early treatment response.

Among *FLT3*-ITD-positive patients achieving induction remission, post-remission HSCT was associated with improved EFS but not OS, with the benefit most evident in the early post-remission period (Section 3.10). This exploratory, unadjusted analysis is subject to treatment-selection and time-related biases and does not establish a causal survival benefit; validation in larger cohorts with data on transplant eligibility, measurable residual disease, and conditioning regimens is warranted.

This study has several strengths. First, the integration of a large TARGET discovery cohort and an independent CHCMU validation cohort provides evidence for consistency of selected genomic associations with IF across different populations. Second, unlike previous studies focusing primarily on individual prognostic biomarkers, we performed multidimensional genomic characterization incorporating recurrent mutations, fusion alterations, and gene interaction patterns. Third, the finding that *FLT3*-ITD retains adverse prognostic significance among patients achieving induction remission, together with the absence of a statistically significant additional prognostic effect among patients with IF, provides new insight into the interpretation of molecular risk factors together with early treatment response in pAML.

Several limitations should be acknowledged. First, the CHCMU cohort had a limited sample size (*n* = 98; 21 IF), particularly for analyses of rare genomic alterations and formal interaction testing; larger multicenter studies are required. Second, this retrospective study lacked functional experiments to directly elucidate the mechanisms of induction resistance; future studies integrating functional assays, single-cell sequencing, and MRD monitoring will be valuable. Third, RNA-seq-based profiling in the CHCMU cohort may not fully capture structural variants and copy-number alterations, and cross-cohort comparisons were restricted to genes robustly detectable in both datasets. Furthermore, short-read transcriptome sequencing may detect structural variants, such as *FLT3* internal tandem duplications, with lower sensitivity than DNA-based sequencing; CHCMU estimates of such alterations should therefore be interpreted cautiously because under-detection may have resulted in lower observed frequencies and greater uncertainty in the estimated associations. Fourth, the higher IF rate (21.4%) in CHCMU likely reflects its inclusion criteria, referral bias, and single-center practice; the same standardized IF definition was applied in both cohorts, supporting the internal validity of the IF–OS comparison. Fifth, salvage treatment after IF was not systematically recorded in the TARGET cohort, and the small number of IF cases precluded cytogenetic regression analyses in CHCMU. In addition, exploratory analyses were not adjusted for multiple comparisons, and only findings replicated across both cohorts were emphasized.

## 5. Conclusions

In conclusion, this study provides a comprehensive clinical and genomic characterization of IF in pAML. IF was associated with poor survival outcomes and distinct molecular features, with *FLT3* mutation showing the most consistent genomic association with IF across the two independent cohorts. Importantly, *FLT3*-ITD remained associated with inferior survival among patients who achieved induction remission, indicating that its adverse prognostic association persisted despite successful initial treatment. Together, these findings highlight the complementary value of early treatment response and baseline molecular characteristics in the assessment of prognostic risk in pAML.

## Figures and Tables

**Table 1:** Univariate and multivariable logistic regression analyses identifying factors associated with IF in the TARGET and CHCMU cohorts.

	TARGET						CHCMU					
	Univariate Analysis			Multivariable Analysis			Univariate Analysis			Multivariable Analysis		
Variable	OR	95% CI	*p*	OR	95% CI	*p*	OR	95% CI	*p*	OR	95% CI	*p*
**Gender**												
male & female	0.528	0.296–0.941	**0.030**	0.471	0.239–0.891	**0.024**	0.541	0.197–1.486	0.233			
**Age at diagnosis**												
	0.971	0.927–1.018	0.222				1.092	0.979–1.219	0.115			
**WBC at diagnosis**												
≤50 vs. >50	2.124	1.209–3.730	**0.009**				1.050	0.331–3.336	0.934			
**BM blasts**												
≤70% vs. >70%	1.690	0.936–3.052	**0.082**				1.029	0.356–2.971	0.958			
**Peripheral blasts**												
≤20% vs. >20%	1.790	0.882–3.632	0.107				0.978	0.293–3.497	0.972			
***ASXL1* mutation**												
No & Yes	2.415	0.791–7.368	0.121				0.720	0.080–6.520	0.770			
***KIT* mutation**												
No & Yes	0.112	0.015–0.817	**0.031**				1.653	0.549–4.977	0.371			
***FLT3* mutation**												
No & Yes	3.615	2.030–6.439	**0.000**	3.180	1.635–6.119	**0.000**	6.091	2.06–18.006	**0.001**	5.361	1.75–16.83	**0.003**
***WT1* mutation**												
No & Yes	2.850	1.519–5.349	**0.001**	2.110	1.004–4.275	**0.042**	3.125	0.878–11.124	**0.079**	1.870	0.43–7.46	0.382
***PTPN11* mutation**												
No & Yes	1.769	0.758–4.131	0.187				1.233	0.122–12.505	0.859			
***KRAS* mutation**												
No & Yes	0.851	0.352–2.053	0.719				0.378	0.045–3.164	0.369			
***NRAS* mutation**												
No & Yes	0.690	0.362–1.316	0.260				2.346	0.830–6.629	0.108			
***CEBPA* mutation**												
No & Yes	0.684	0.206–2.273	0.535				0.518	0.107–2.502	0.413			
***RUNX1* mutation**												
No & Yes	1.590	0.352–7.185	0.547				1.233	0.122–12.505	0.859			
***NPM1* mutation**												
No & Yes	0.135	0.018–0.988	**0.049**				0	NE	0.999			
***TP53* mutation**												
No & Yes	1.380	0.169–11.237	0.764				3.8	0.228–63.449	0.353			
***IDH2* mutation**												
No & Yes	0	NE	0.998				0	NE	0.999			

Note: Odds ratios and *p* values were estimated by univariate and multivariable logistic regression. For each variable, the odds ratio compares the second category with the first (reference category). Univariate analyses in the TARGET cohort were performed in the 659 patients with genomic profiling data; multivariable analysis was performed on 617 patients with complete data for all model covariates. Only variables with *p* < 0.05 in the TARGET multivariable model are shown; the full TARGET model additionally included WBC > 50 × 10^9^/L, BM blasts >70%, *KIT* mutation, and *NPM1* mutation (all *p* > 0.05). The CHCMU multivariable model included *FLT3* and *WT1* mutations. Age did not meet the univariate screening threshold (*p* < 0.10) and was instead included in the forced-entry sensitivity analysis together with sex and WBC count. WBC, white blood cell count; BM, bone marrow; NE, not estimable (zero event cell or complete separation). *p* values < 0.05 are shown in bold.

## Data Availability

TARGET program data are publicly available through the GDC data Portal (https://portal.gdc.cancer.gov/). CHCMU cohort data are not publicly available due to privacy restrictions but can be requested from the corresponding author. All analysis scripts and additional information are available upon reasonable request.

## Supplementary Materials

The following supporting information can be downloaded at: https://www.mdpi.com/article/10.3390/cancers18203280/s1, Figure S1: Landmark sensitivity analyses of the association between induction failure (IF) and overall survival (OS) in the TARGET and CHCMU cohorts. HRs of IF for OS were estimated at landmark time points of 60, 90, 120, 150, and 180 days after diagnosis in both cohorts; Figure S2: Prognostic impact of IF across treatment arms. (A–C) Kaplan–Meier curves comparing OS between patients with and without IF within (A) Arm A (ADE), (B) Arm B (ADE + bortezomib), and (C) Arm C (ADE + sorafenib). (D) Forest plot showing the interaction effects between IF status and treatment arm assignment on OS based on Cox proportional hazards regression, with Arm A as the reference treatment arm; Figure S3: OS according to sorafenib-containing therapy (Arm C) by *FLT3*-ITD and WBC status. Kaplan–Meier curves comparing Arm C (sorafenib) with Arms A/B (no sorafenib) among (A) all *FLT3*-ITD-positive patients; (B) patients with high WBC; (C) patients with both high WBC and *FLT3*-ITD; (D) *FLT3*-ITD-positive patients with low WBC; Figure S4: Significant pairwise genetic interaction patterns in non-IF patients of the TARGET cohort; Figure S5: Pairwise genetic interaction patterns in the CHCMU cohort. Genetic interaction analysis based on pathogenic and likely pathogenic variants (Levels 1 and 2) identified in the CHCMU validation cohort; Figure S6: Fusion gene distribution in the CHCMU cohort. Circos plots illustrating chromosomal rearrangements and fusion gene distributions in IF (A) and non-IF (B) patients from the CHCMU cohort; Figure S7: Factors associated with IF in the TARGET discovery cohort. Forest plot showing odds ratios (ORs) and 95% confidence intervals (CIs) for clinical and genomic factors identified by multivariable logistic regression analysis; Figure S8: Multivariable logistic regression analysis of IF in the CHCMU cohort. Forest plot showing clinical and genomic factors associated with IF in the CHCMU validation cohort; Figure S9: LASSO logistic regression and bootstrap stability analysis in the TARGET cohort (*n* = 659). (A) Cross-validation curve of the LASSO model; (B) variable selection frequencies across 1000 bootstrap resamples; Figure S10: IF rates by cytogenetic class in the CHCMU and TARGET cohorts. IF rates were compared between cohorts for favorable-risk karyotypes (t(8;21) and inv(16)), *KMT2A*-rearranged, normal karyotype, and other abnormalities (Fisher’s exact test). * *p* < 0.05; ** *p* < 0.01; † *p* < 0.10; Figure S11: Prognostic association of *FLT3*-ITD according to induction response status. (A) Kaplan–Meier curves comparing OS between *FLT3*-ITD–positive and *FLT3*-ITD–negative patients within the IF subgroup; (B) Kaplan–Meier curves comparing OS between *FLT3*-ITD–positive and *FLT3*-ITD–negative patients within the non-IF subgroup; Figure S12: Interaction between *NPM1* mutation and *FLT3*-ITD on OS and IF in the TARGET cohort. (A) Forest plot showing hazard ratios for *FLT3*-ITD on OS stratified by *NPM1* genotype and the *NPM1* × *FLT3*-ITD interaction term; (B) IF rates by *NPM1*/*FLT3*-ITD genotype; Figure S13: Association of HSCT with outcomes in *FLT3*-ITD-positive patients without IF. (A) Kaplan–Meier curves comparing EFS between *FLT3*-ITD–positive patients without IF who underwent HSCT in first complete remission and those who did not; (B) Kaplan–Meier curves comparing OS between *FLT3*-ITD–positive patients without IF who underwent HSCT in first complete remission and those who did not; Table S1: Overview of the induction and intensification regimens used in the TARGET (COG AAML1031) and CHCMU cohorts (as described in references [9,44]); Table S2: (A) Baseline characteristics of pAML patients in the TARGET cohort. (B) Baseline characteristics of pAML patients in the CHCMU cohort; Table S3: Time-dependent hazard ratios of IF for OS; Table S4: Clinical characteristics of patients with pathogenic mutations (Levels 1 and 2) in the CHCMU validation cohort; Table S5: (A) Significant co-mutation and mutual exclusivity analysis in the TARGET cohort. (B) Significant co-mutation and mutual exclusivity in IF patients of the TARGET cohort. (C) Significant co-mutation and mutual exclusivity in non-IF patients of the TARGET cohort. (D) Genes with differential mutation enrichment between IF and non-IF patients in the TARGET cohort. (E) Complete pairwise co-mutation and mutual exclusivity results in the TARGET cohort. (F) Co-mutation and mutual exclusivity results in the CHCMU cohort. (G) Genes with differential mutation enrichment in the CHCMU cohort; Table S6: Clinical characteristics of patients with fusion genes in the CHCMU validation cohort; Table S7: (A) Gene fusions identified in the TARGET cohort. (B) Gene fusions identified in the CHCMU cohort. (C) Association between gene fusions and IF in the TARGET cohort. (D) Association between gene fusions and IF in the CHCMU cohort; Table S8: Univariate comparison of cytogenetic aberrations between IF and non-IF patients in the TARGET cohort; Table S9: Multivariable logistic regression of cytogenetic and clinical factors associated with IF in the TARGET cohort; Table S10: Extended multivariable model including cytogenetic covariates assessing whether cytogenetic risk confounds the *FLT3*–IF association in the TARGET cohort; Table S11: (A) Interaction between *NPM1* mutation and *FLT3*-ITD on OS in the TARGET cohort. (B) Interaction between *NPM1* mutation and *FLT3*-ITD on IF in the TARGET cohort; Table S12: Association of salvage therapy and HSCT with mutation status of key genes (*FLT3*, *WT1*, *KIT*, *NRAS*, and *CEBPA*) among the IF patients in the CHCMU cohort.
