# Supplementary material for: Genomic Determinants of Induction Failure and Persistent Prognostic Risk of FLT3-ITD in Pediatric Acute Myeloid Leukemia
Source: Cancers (Basel). 2026 Oct 12;18(20):3280. doi: 10.3390/cancers18203280 (PMC13702683; doi:10.3390/cancers18203280)
Supplement: Supplementary file 1 [file cancers-18-03280-s001.zip › Figure S1.pdf]

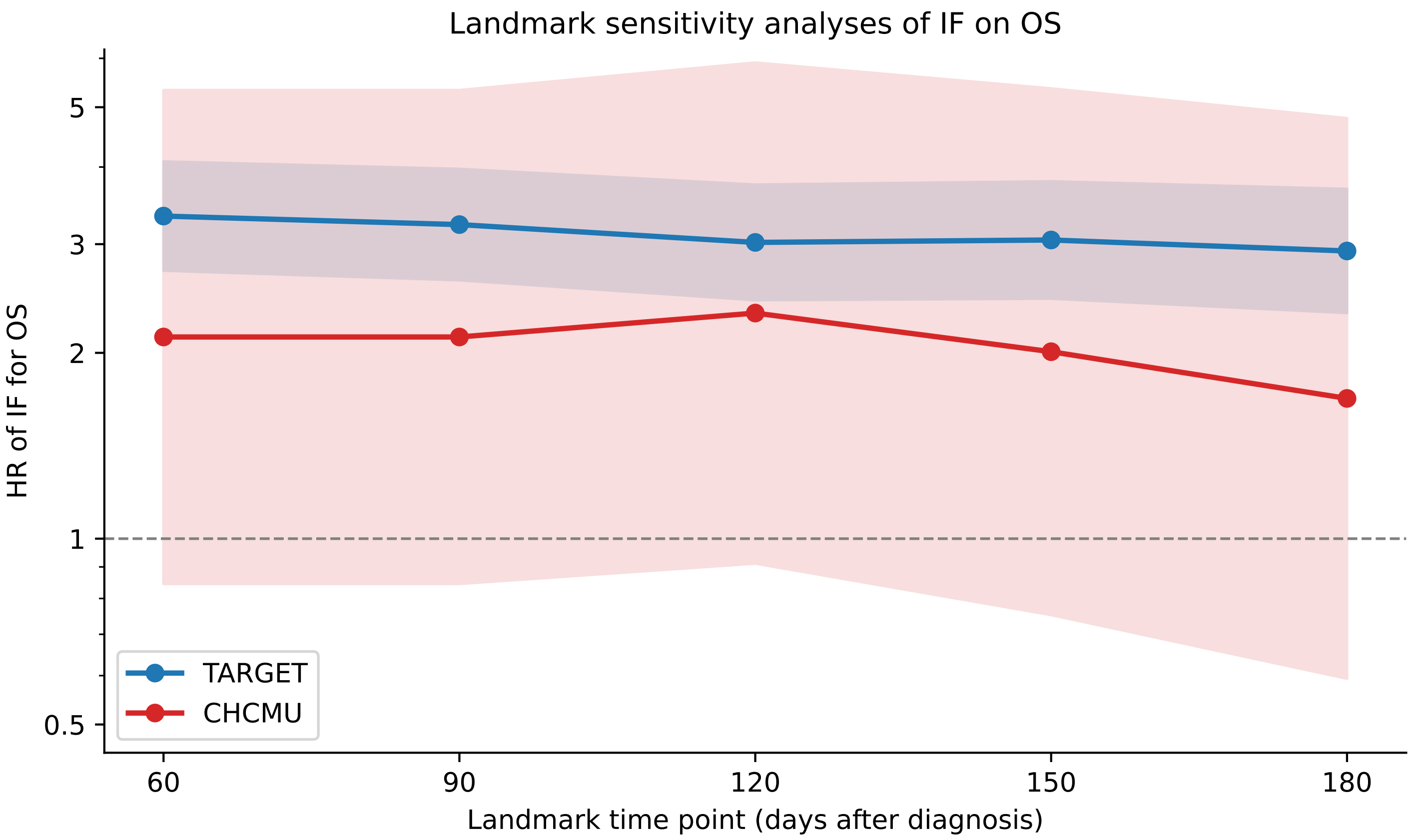

Landmark sensitivity analyses of IF on OS
HR of IF for OS
5
3
2
1
0.5
60
90
120
150
180
Landmark time point (days after diagnosis)
TARGET
CHCMU
