# Supplementary material for: Genomic Determinants of Induction Failure and Persistent Prognostic Risk of FLT3-ITD in Pediatric Acute Myeloid Leukemia
Source: Cancers (Basel). 2026 Oct 12;18(20):3280. doi: 10.3390/cancers18203280 (PMC13702683; doi:10.3390/cancers18203280)
Supplement: Supplementary file 1 [file cancers-18-03280-s001.zip › Figure S10.pdf]

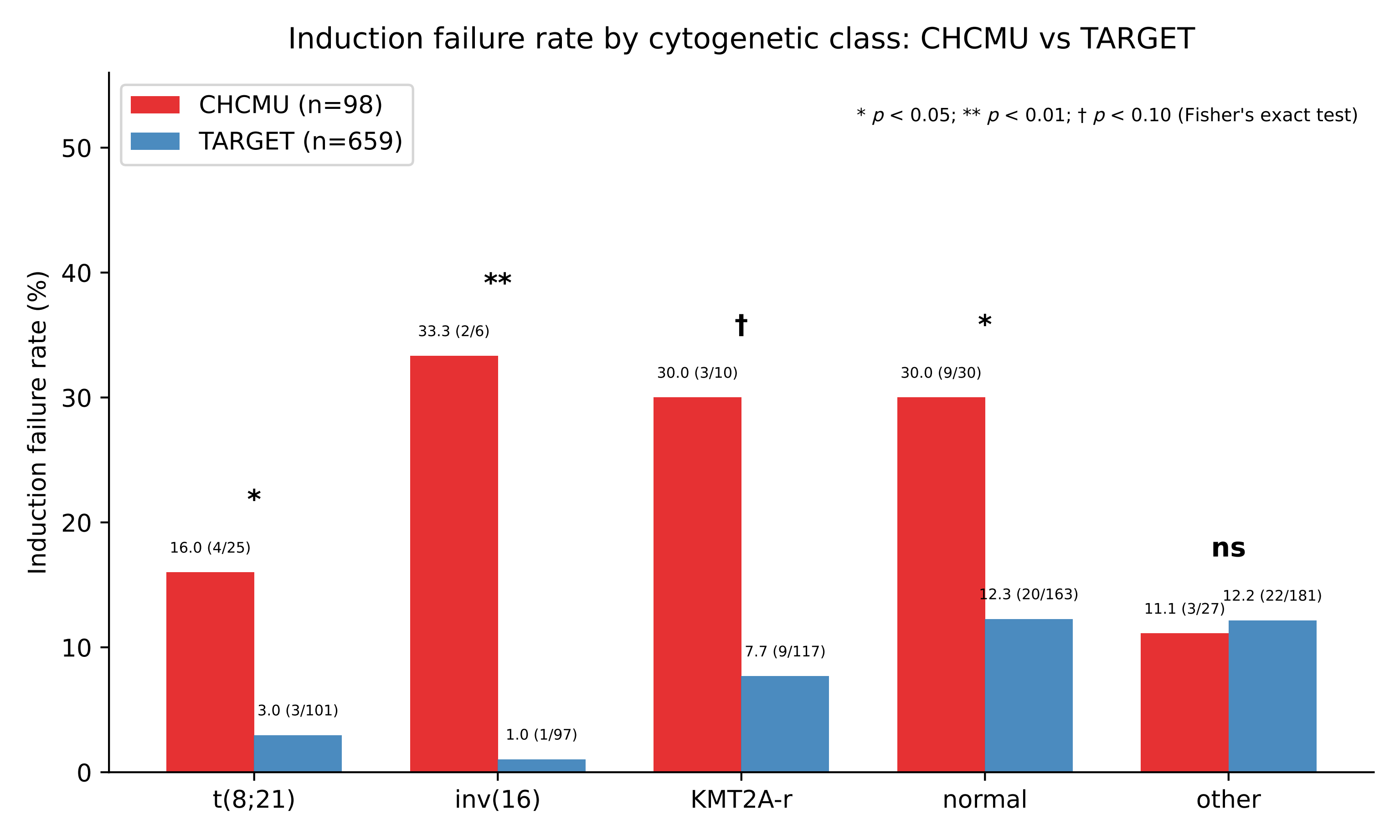

Induction failure rate by cytogenetic class: CHCMU vs TARGET
CHCMU (n=98)
TARGET (n=659)
* p < 0.05; ** p < 0.01; † p < 0.10 (Fisher's exact test)
Induction failure rate (%)
0
10
20
30
40
50
16.0 (4/25)
3.0 (3/101)
*
33.3 (2/6)
1.0 (1/97)
**
30.0 (3/10)
7.7 (9/117)
†
30.0 (9/30)
12.3 (20/163)
*
11.1 (3/27)
12.2 (22/181)
ns
t(8;21)
inv(16)
KMT2A-r
normal
other
