# Supplementary material for: Genomic Determinants of Induction Failure and Persistent Prognostic Risk of FLT3-ITD in Pediatric Acute Myeloid Leukemia
Source: Cancers (Basel). 2026 Oct 12;18(20):3280. doi: 10.3390/cancers18203280 (PMC13702683; doi:10.3390/cancers18203280)
Supplement: Supplementary file 1 [file cancers-18-03280-s001.zip › Figure S11.pdf]

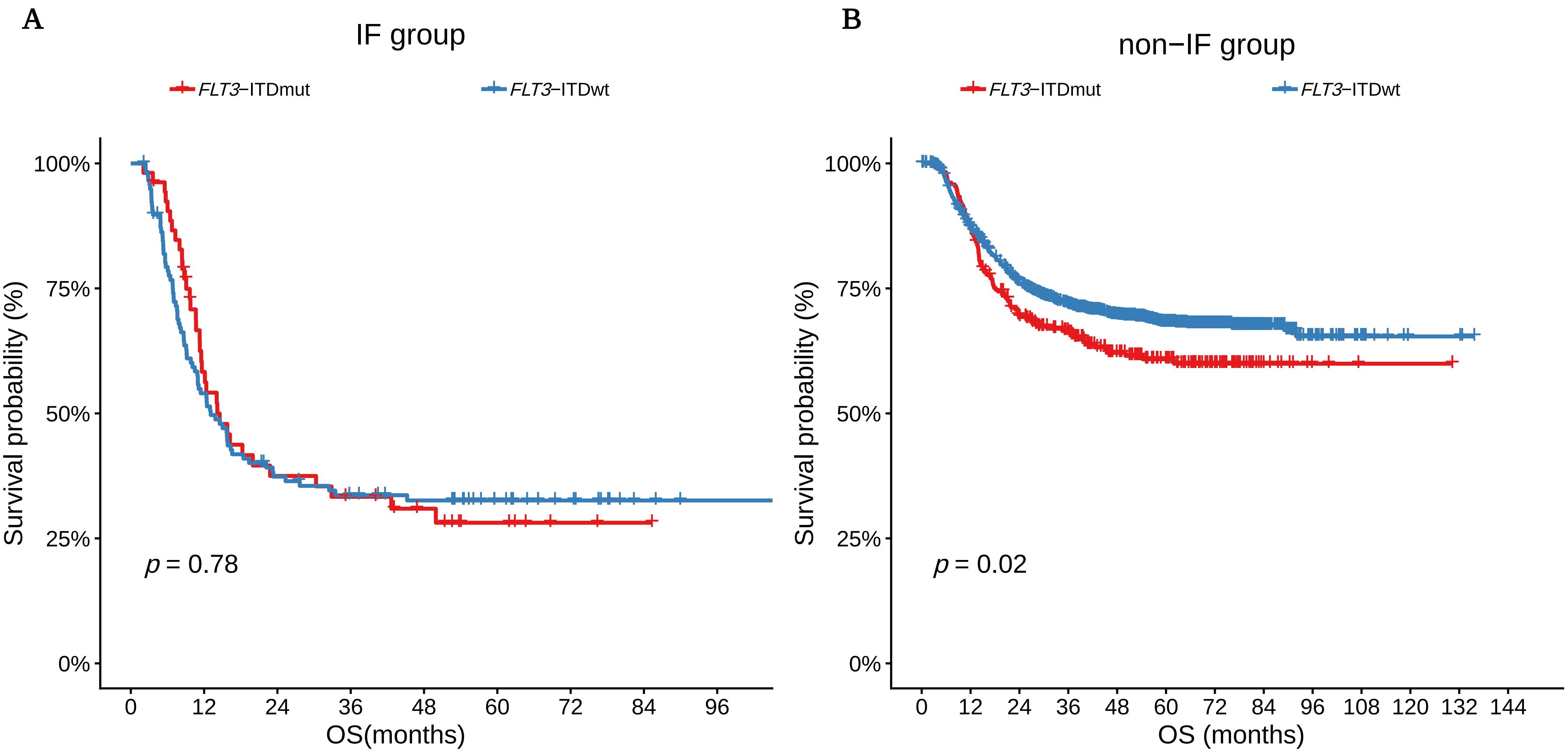

A
IF group
FLT3−ITDmut
FLT3−ITDwt
100%
75%
50%
25%
0%
Survival probability (%)
p = 0.78
0
12
24
36
48
60
72
84
96
OS(months)
B
non−IF group
FLT3−ITDmut
FLT3−ITDwt
100%
75%
50%
25%
0%
Survival probability (%)
p = 0.02
0
12
24
36
48
60
72
84
96
108
120
132
144
OS (months)
