# Supplementary material for: Genomic Determinants of Induction Failure and Persistent Prognostic Risk of FLT3-ITD in Pediatric Acute Myeloid Leukemia
Source: Cancers (Basel). 2026 Oct 12;18(20):3280. doi: 10.3390/cancers18203280 (PMC13702683; doi:10.3390/cancers18203280)
Supplement: Supplementary file 1 [file cancers-18-03280-s001.zip › Figure S12.pdf]

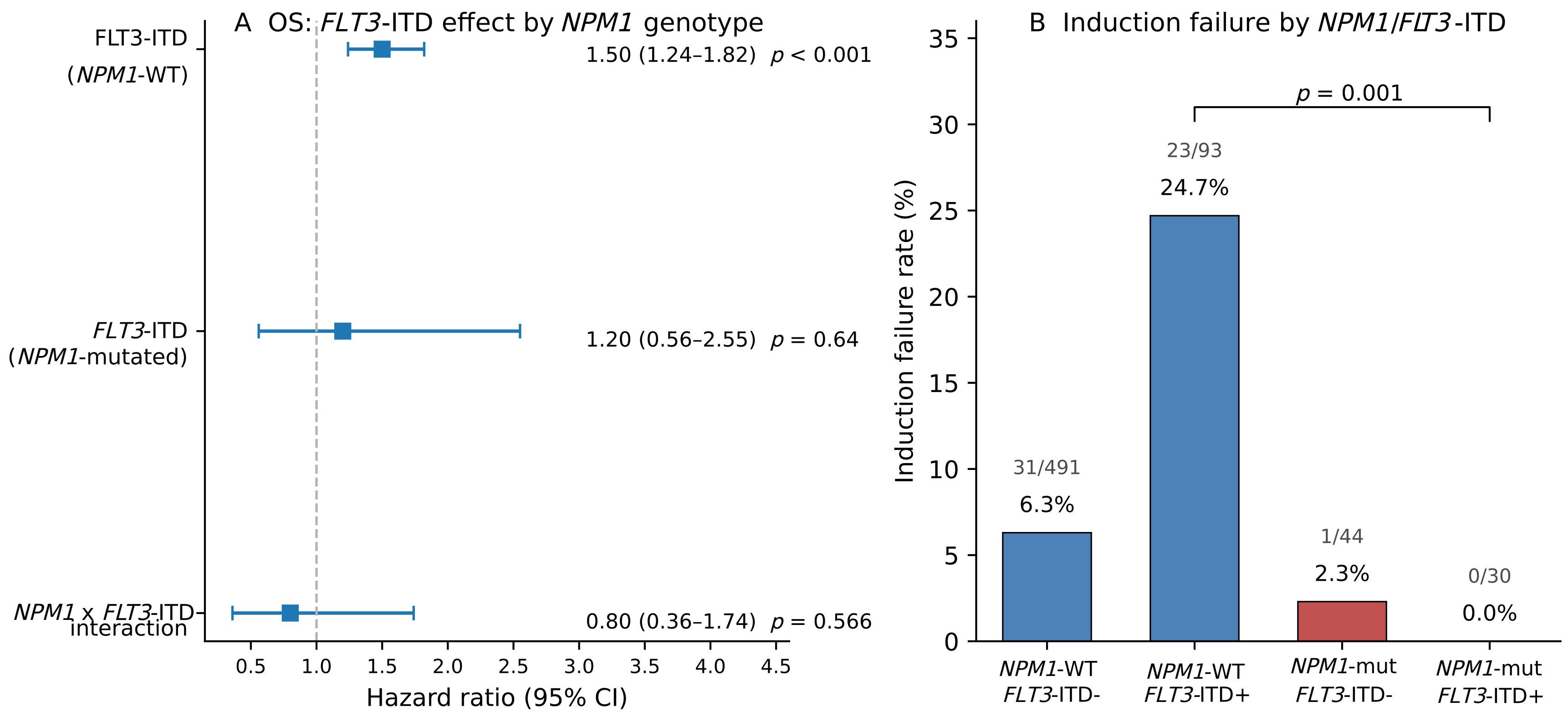
A OS: FLT3-ITD effect by NPM1 genotype
FLT3-ITD
(NPM1-WT)
FLT3-ITD
(NPM1-mutated)
NPM1 x FLT3-ITD
interaction
1.50 (1.24–1.82) p < 0.001
1.20 (0.56–2.55) p = 0.64
0.80 (0.36–1.74) p = 0.566
0.5
1.0
1.5
2.0
2.5
3.0
3.5
4.0
4.5
Hazard ratio (95% CI)
B Induction failure by NPM1/FLT3-ITD
Induction failure rate (%)
0
5
10
15
20
25
30
35
p = 0.001
31/491
6.3%
23/93
24.7%
1/44
2.3%
0/30
0.0%
NPM1-WT
FLT3-ITD-
NPM1-WT
FLT3-ITD+
NPM1-mut
FLT3-ITD-
NPM1-mut
FLT3-ITD+
