# Supplementary material for: Genomic Determinants of Induction Failure and Persistent Prognostic Risk of FLT3-ITD in Pediatric Acute Myeloid Leukemia
Source: Cancers (Basel). 2026 Oct 12;18(20):3280. doi: 10.3390/cancers18203280 (PMC13702683; doi:10.3390/cancers18203280)
Supplement: Supplementary file 1 [file cancers-18-03280-s001.zip › Figure S13.pdf]

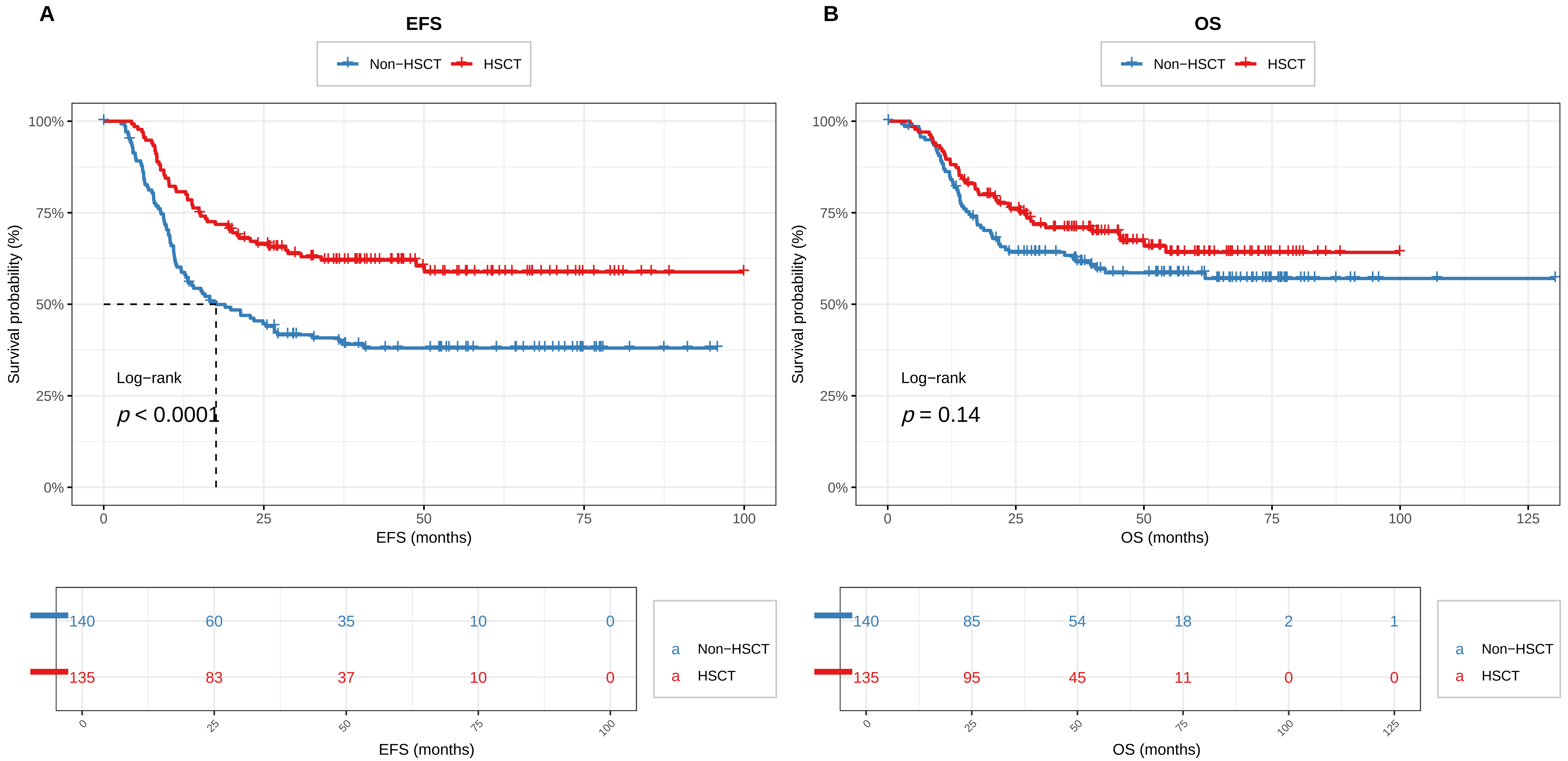
A
EFS
Non−HSCT
HSCT
100%
75%
50%
25%
0%
Survival probability (%)
Log−rank
p < 0.0001
0
25
50
75
100
EFS (months)
140
60
35
10
0
135
83
37
10
0
a Non−HSCT
a HSCT
EFS (months)
B
OS
Non−HSCT
HSCT
Survival probability (%)
Log−rank
p = 0.14
125
OS (months)
140
85
54
18
2
1
135
95
45
11
0
0
a Non−HSCT
a HSCT
OS (months)
