# Supplementary material for: Genomic Determinants of Induction Failure and Persistent Prognostic Risk of FLT3-ITD in Pediatric Acute Myeloid Leukemia
Source: Cancers (Basel). 2026 Oct 12;18(20):3280. doi: 10.3390/cancers18203280 (PMC13702683; doi:10.3390/cancers18203280)
Supplement: Supplementary file 1 [file cancers-18-03280-s001.zip › Figure S2.pdf]

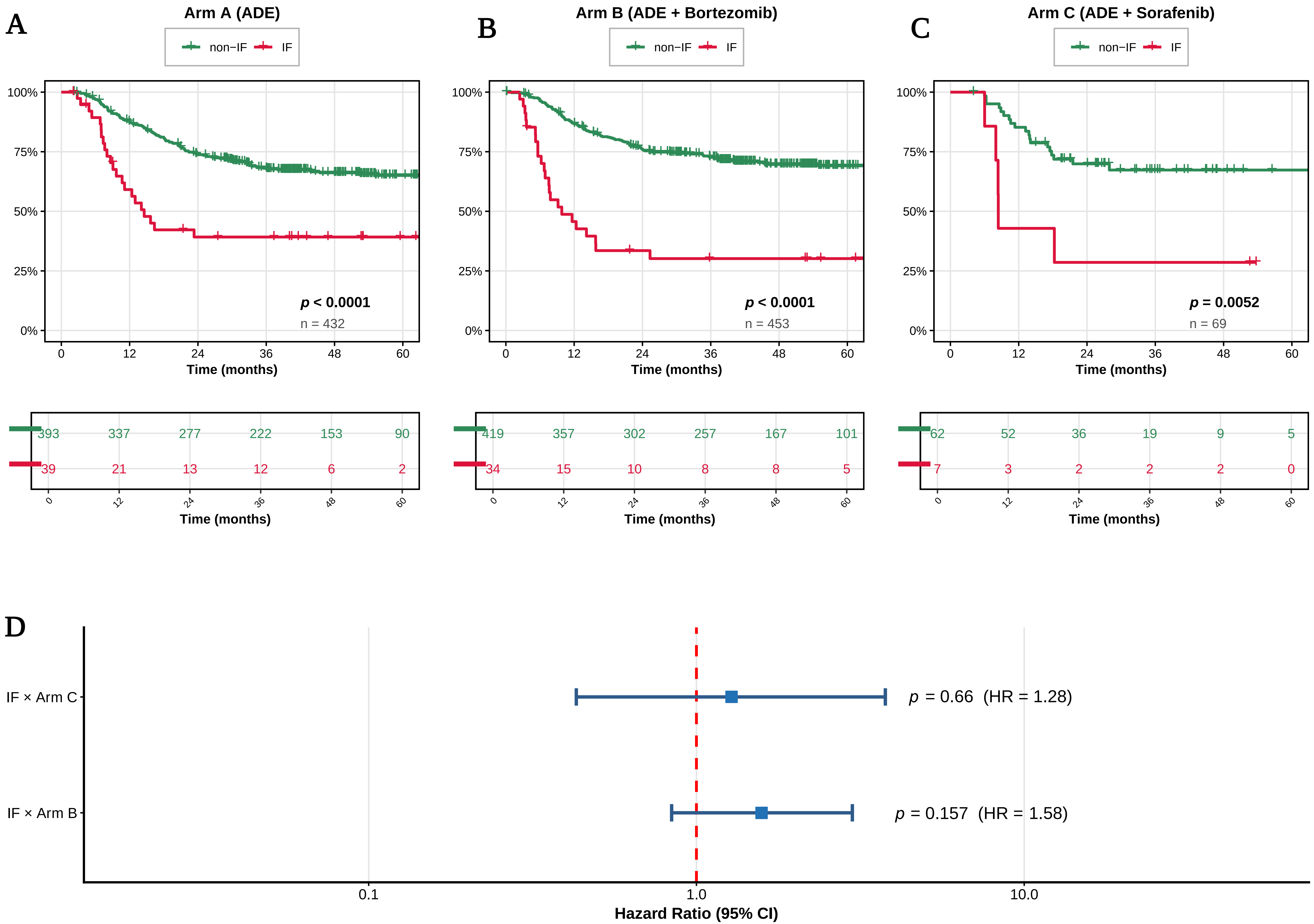

A
Arm A (ADE)
non−IF
IF
100%
75%
50%
25%
0%
p < 0.0001
n = 432
Time (months)
393 337 277 222 153 90
39 21 13 12 6 2
B
Arm B (ADE + Bortezomib)
p < 0.0001
n = 453
419 357 302 257 167 101
34 15 10 8 8 5
C
Arm C (ADE + Sorafenib)
p = 0.0052
n = 69
62 52 36 19 9 5
7 3 2 2 2 0
0 12 24 36 48 60
D
IF × Arm C
IF × Arm B
p = 0.66 (HR = 1.28)
p = 0.157 (HR = 1.58)
0.1
1.0
10.0
Hazard Ratio (95% CI)
