# Supplementary material for: Genomic Determinants of Induction Failure and Persistent Prognostic Risk of FLT3-ITD in Pediatric Acute Myeloid Leukemia
Source: Cancers (Basel). 2026 Oct 12;18(20):3280. doi: 10.3390/cancers18203280 (PMC13702683; doi:10.3390/cancers18203280)
Supplement: Supplementary file 1 [file cancers-18-03280-s001.zip › Figure S3.pdf]

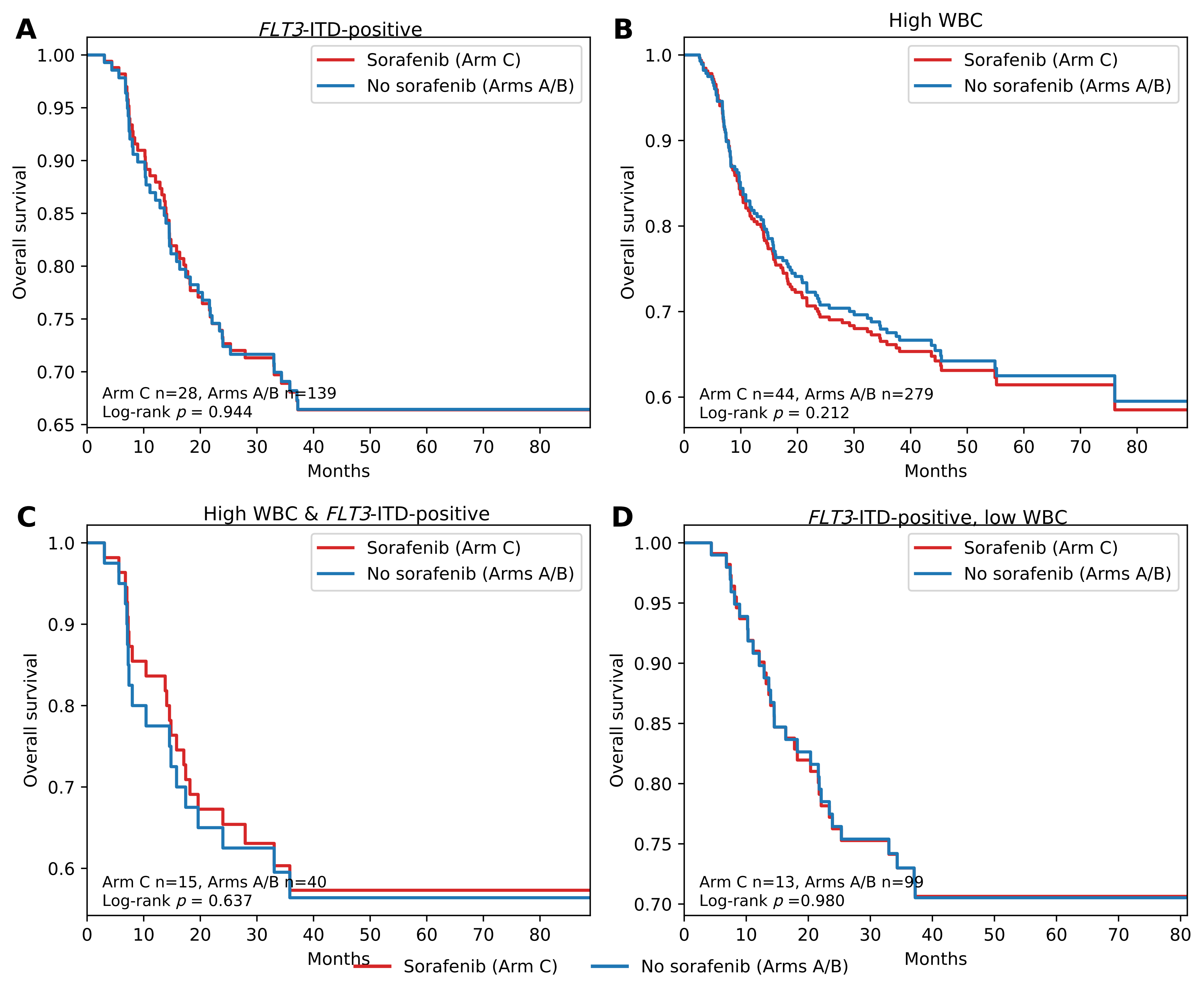

A
FLT3-ITD-positive
Sorafenib (Arm C)
No sorafenib (Arms A/B)
Overall survival
1.00
0.95
0.90
0.85
0.80
0.75
0.70
0.65
Arm C n=28, Arms A/B n=139
Log-rank *p* = 0.944
0 10 20 30 40 50 60 70 80
Months
B
High WBC
Sorafenib (Arm C)
No sorafenib (Arms A/B)
Overall survival
1.0
0.9
0.8
0.7
0.6
Arm C n=44, Arms A/B n=279
Log-rank *p* = 0.212
0 10 20 30 40 50 60 70 80
Months
C
High WBC & *FLT3*-ITD-positive
Sorafenib (Arm C)
No sorafenib (Arms A/B)
Overall survival
1.0
0.9
0.8
0.7
0.6
Arm C n=15, Arms A/B n=40
Log-rank *p* = 0.637
0 10 20 30 40 50 60 70 80
Months
D
FLT3-ITD-positive, low WBC
Sorafenib (Arm C)
No sorafenib (Arms A/B)
Overall survival
1.00
0.95
0.90
0.85
0.80
0.75
0.70
Arm C n=13, Arms A/B n=99
Log-rank *p* =0.980
0 10 20 30 40 50 60 70 80
Months
Sorafenib (Arm C)
No sorafenib (Arms A/B)
