# Supplementary material for: Genomic Determinants of Induction Failure and Persistent Prognostic Risk of FLT3-ITD in Pediatric Acute Myeloid Leukemia
Source: Cancers (Basel). 2026 Oct 12;18(20):3280. doi: 10.3390/cancers18203280 (PMC13702683; doi:10.3390/cancers18203280)
Supplement: Supplementary file 1 [file cancers-18-03280-s001.zip › Figure S4.pdf]

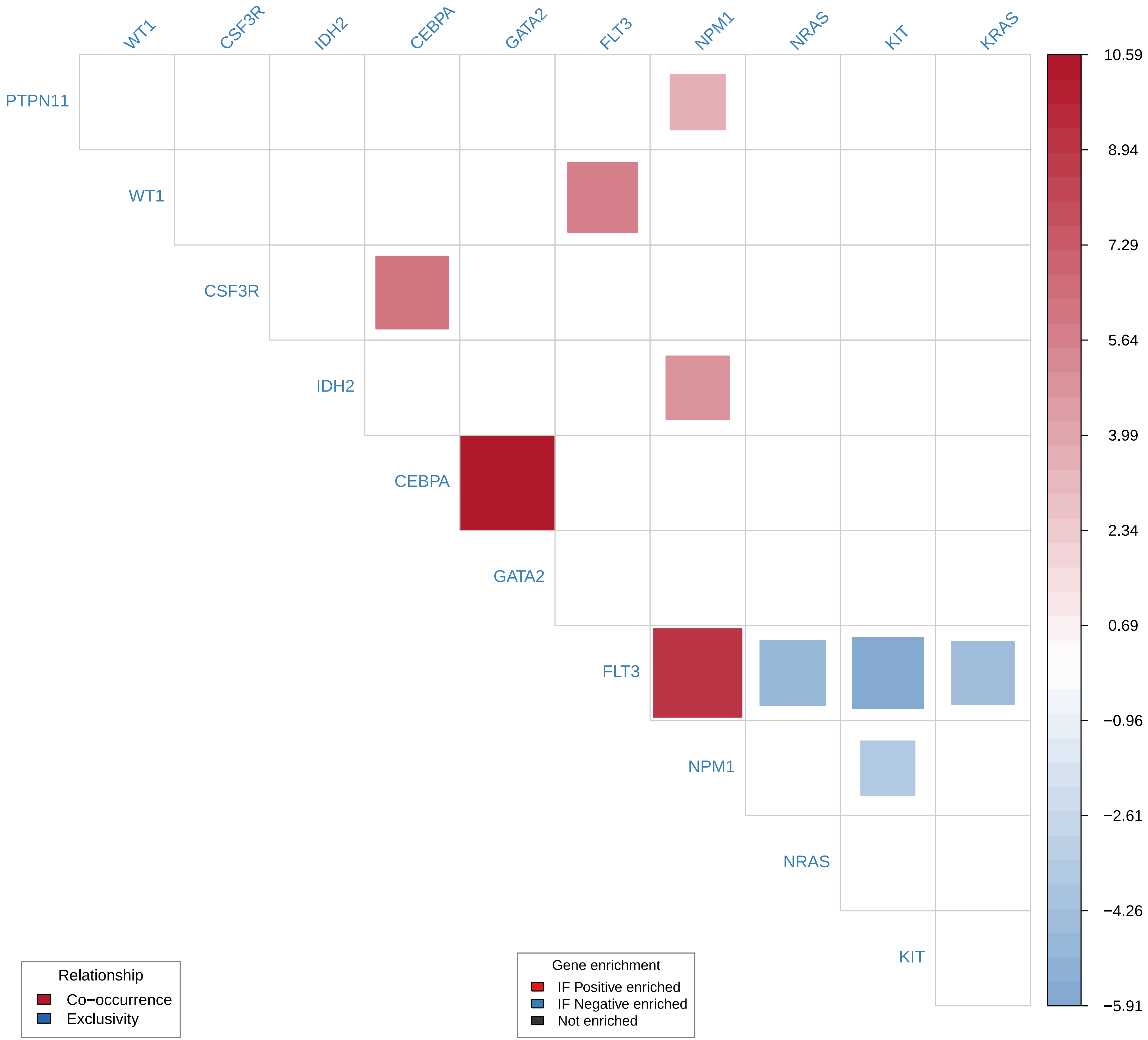

WT1
CSF3R
IDH2
CEBPA
GATA2
FLT3
NPM1
NRAS
KIT
KRAS
PTPN11
WT1
CSF3R
IDH2
CEBPA
GATA2
FLT3
NPM1
NRAS
KIT
10.59
8.94
7.29
5.64
3.99
2.34
0.69
−0.96
−2.61
−4.26
−5.91
Relationship
Co−occurrence
Exclusivity
Gene enrichment
IF Positive enriched
IF Negative enriched
Not enriched
