# Supplementary material for: Genomic Determinants of Induction Failure and Persistent Prognostic Risk of FLT3-ITD in Pediatric Acute Myeloid Leukemia
Source: Cancers (Basel). 2026 Oct 12;18(20):3280. doi: 10.3390/cancers18203280 (PMC13702683; doi:10.3390/cancers18203280)
Supplement: Supplementary file 1 [file cancers-18-03280-s001.zip › Figure S5.pdf]

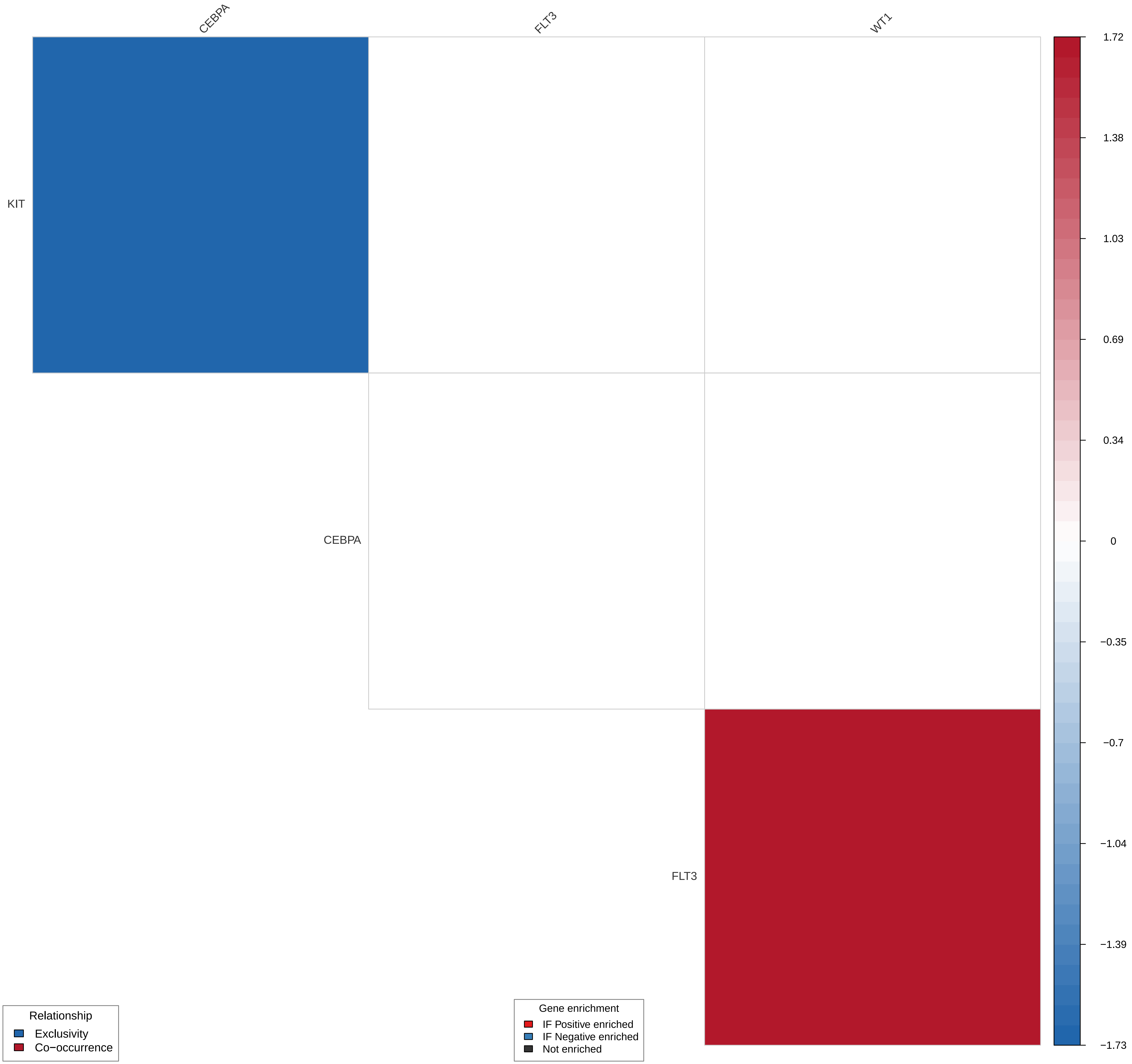

CEBPA
FLT3
WT1
KIT
CEBPA
FLT3
1.72
1.38
1.03
0.69
0.34
0
−0.35
−0.7
−1.04
−1.39
−1.73
Relationship
Exclusivity
Co−occurrence
Gene enrichment
IF Positive enriched
IF Negative enriched
Not enriched
