# Supplementary material for: Genomic Determinants of Induction Failure and Persistent Prognostic Risk of FLT3-ITD in Pediatric Acute Myeloid Leukemia
Source: Cancers (Basel). 2026 Oct 12;18(20):3280. doi: 10.3390/cancers18203280 (PMC13702683; doi:10.3390/cancers18203280)
Supplement: Supplementary file 1 [file cancers-18-03280-s001.zip › Figure S6.pdf]

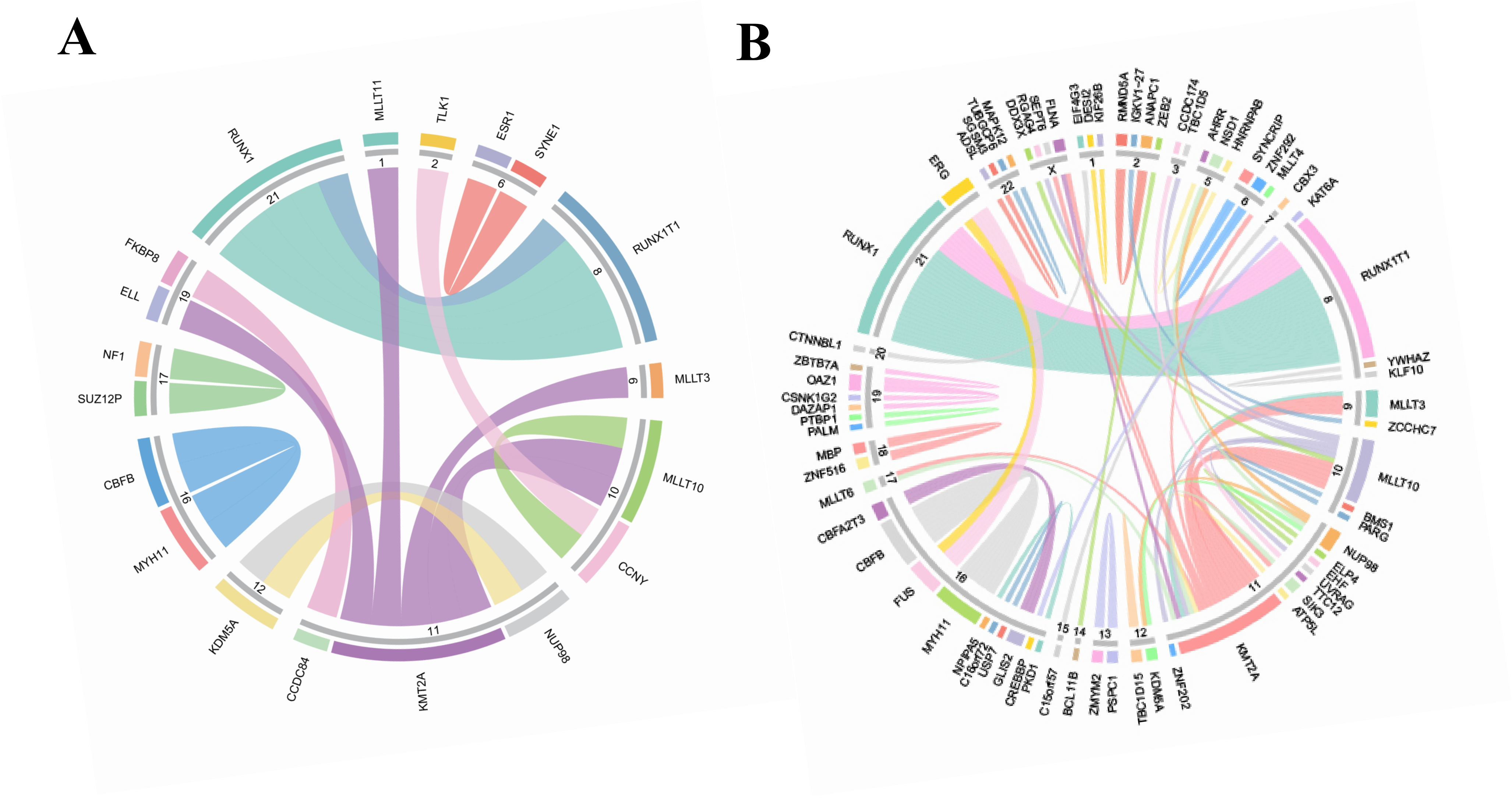
A
RUNX1
MLLT11
TLK1
ESR1
SYNE1
RUNX1T1
MLLT3
MLLT10
CCNY
NUP98
KMT2A
CCDC84
KDM5A
MYH11
CBFB
SUZ12P
NF1
ELL
FKBP8
B
ERG
RUNX1
CTNNBL1
ZBTB7A
OAZ1
CSNK1G2
DAZAP1
PTBP1
PALM
MBP
ZNF516
MLLT6
CBFA2T3
CBFB
FUS
MYH11
GLIS2
CREBBP
PKD1
C15orf57
BCL11B
ZMYM2
PSPC1
TBC1D15
KDM5A
ZNF202
KMT2A
NUP98
BMS1
PARG
MLLT10
ZCCHC7
MLLT3
YWHAZ
KLF10
RUNX1T1
KAT6A
CBX3
MLLT4
SYNCRIP
HNRNPAB
NSD1
AHRR
CCDC174
TBC1D5
ZEB2
ANAPC1
IGKV1-27
RMND5A
KIF26B
DESI2
EIF4G3
FLNA
DDX3X
MAPK12
USP7
