# Supplementary material for: Genomic Determinants of Induction Failure and Persistent Prognostic Risk of FLT3-ITD in Pediatric Acute Myeloid Leukemia
Source: Cancers (Basel). 2026 Oct 12;18(20):3280. doi: 10.3390/cancers18203280 (PMC13702683; doi:10.3390/cancers18203280)
Supplement: Supplementary file 1 [file cancers-18-03280-s001.zip › Figure S7.pdf]

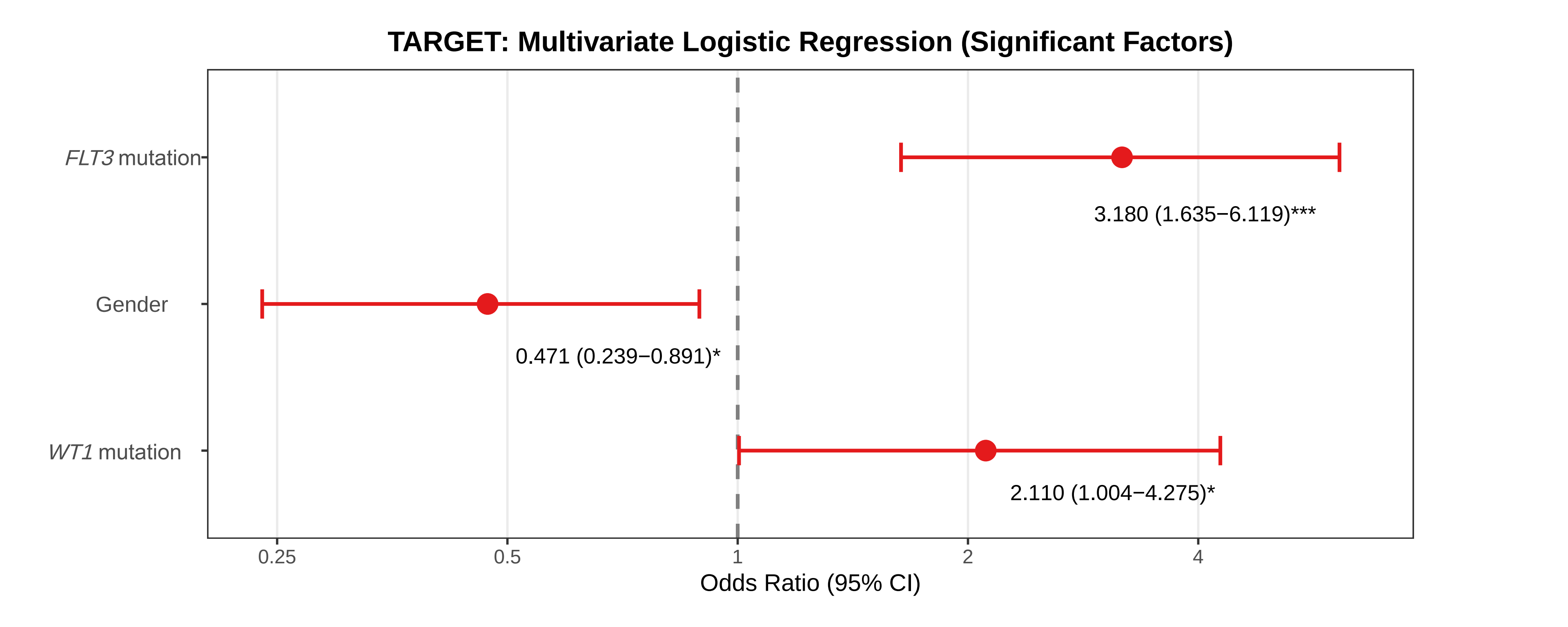

TARGET: Multivariate Logistic Regression (Significant Factors)
FLT3 mutation
Gender
WT1 mutation
3.180 (1.635−6.119)***
0.471 (0.239−0.891)*
2.110 (1.004−4.275)*
0.25
0.5
1
2
4
Odds Ratio (95% CI)
