# Supplementary material for: Genomic Determinants of Induction Failure and Persistent Prognostic Risk of FLT3-ITD in Pediatric Acute Myeloid Leukemia
Source: Cancers (Basel). 2026 Oct 12;18(20):3280. doi: 10.3390/cancers18203280 (PMC13702683; doi:10.3390/cancers18203280)
Supplement: Supplementary file 1 [file cancers-18-03280-s001.zip › Figure S8.pdf]

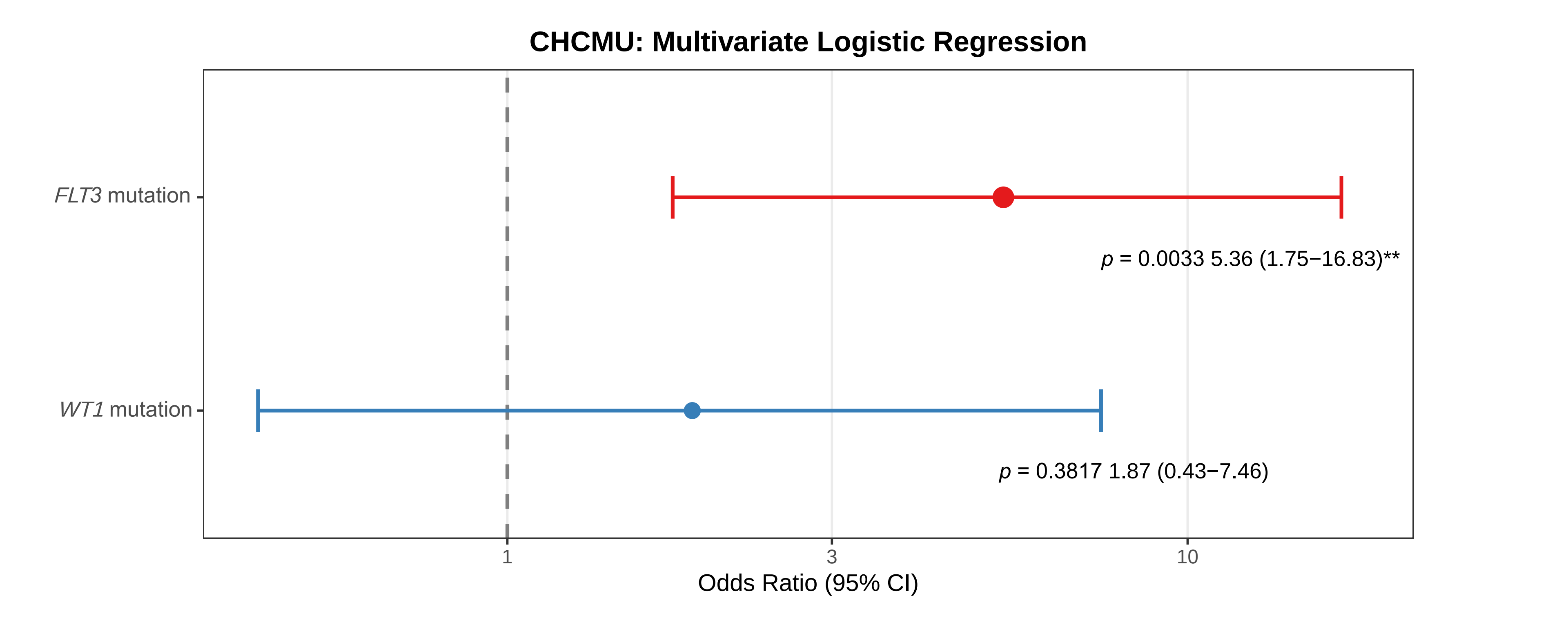
CHCMU: Multivariate Logistic Regression
FLT3 mutation
WT1 mutation
p = 0.0033 5.36 (1.75−16.83)**
p = 0.3817 1.87 (0.43−7.46)
1
3
10
Odds Ratio (95% CI)
