# Supplementary material for: Genomic Determinants of Induction Failure and Persistent Prognostic Risk of FLT3-ITD in Pediatric Acute Myeloid Leukemia
Source: Cancers (Basel). 2026 Oct 12;18(20):3280. doi: 10.3390/cancers18203280 (PMC13702683; doi:10.3390/cancers18203280)
Supplement: Supplementary file 1 [file cancers-18-03280-s001.zip › Figure S9.pdf]

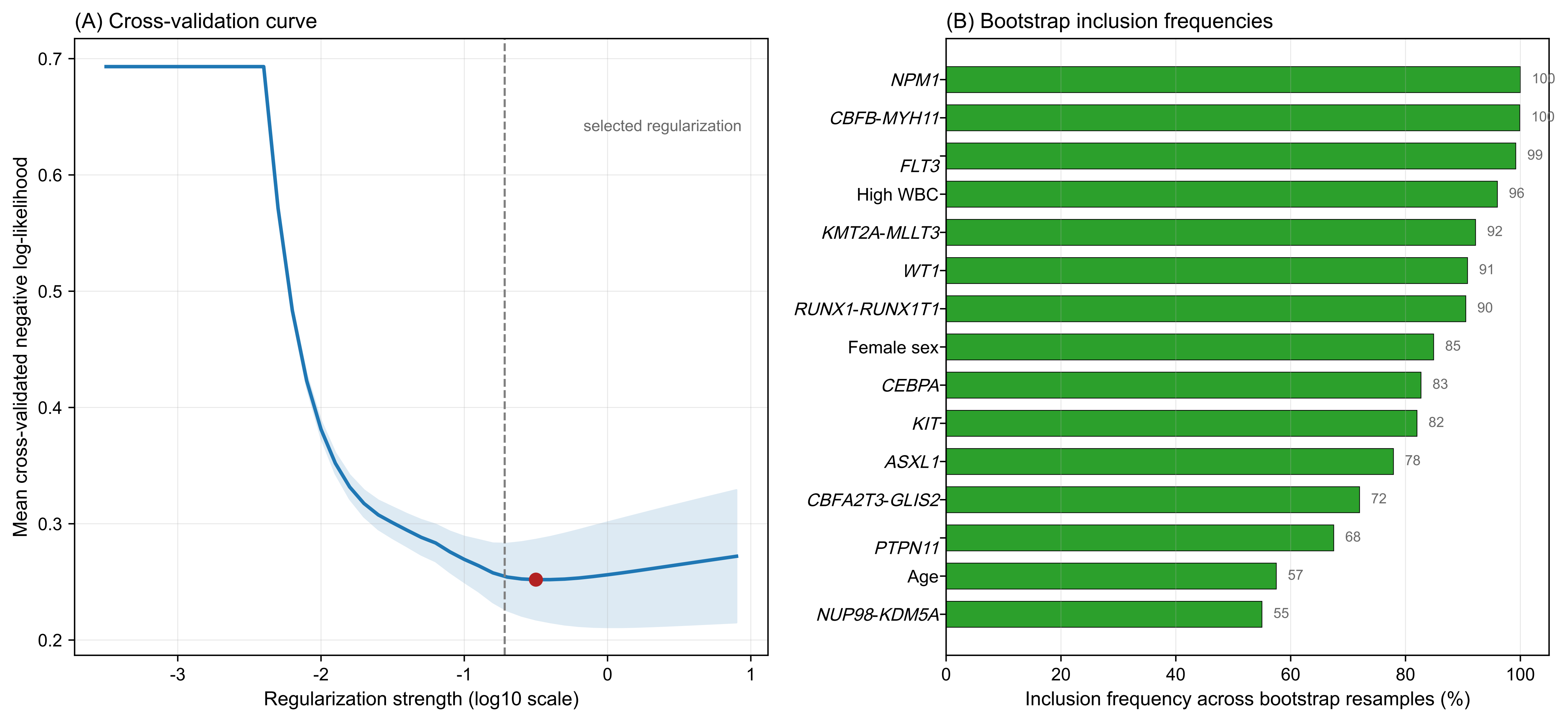

(A) Cross-validation curve
selected regularization
Mean cross-validated negative log-likelihood
0.7
0.6
0.5
0.4
0.3
0.2
-3
-2
-1
0
1
Regularization strength (log10 scale)
(B) Bootstrap inclusion frequencies
NPM1
CBFB-MYH11
FLT3
High WBC
KMT2A-MLLT3
WT1
RUNX1-RUNX1T1
Female sex
CEBPA
KIT
ASXL1
CBFA2T3-GLIS2
PTPN11
Age
NUP98-KDM5A
100
100
99
96
92
91
90
85
83
82
78
72
68
57
55
0
20
40
60
80
100
Inclusion frequency across bootstrap resamples (%)
