# Supplementary material for: Genomic Determinants of Induction Failure and Persistent Prognostic Risk of FLT3-ITD in Pediatric Acute Myeloid Leukemia
Source: Cancers (Basel). 2026 Oct 12;18(20):3280. doi: 10.3390/cancers18203280 (PMC13702683; doi:10.3390/cancers18203280)
Supplement: Supplementary file 1 [file cancers-18-03280-s001.zip › Table S1.pdf]

Table S1. Overview of the induction and intensification regimens used in the TARGET (COG AAML1031) and CHCMU cohorts

**Treatment element**	**Arm A (standard chemotherapy; *n* = 432)**	**Arm B (standard chemotherapy + bortezomib; *n* = 453)**	**Arm C (standard chemotherapy + sorafenib; *n* = 69)**	**CHCMU cohort (*n* = 98)**
**Patient assignment**	Randomized (Arm A vs. Arm B)	Randomized (Arm A vs. Arm B)	Restricted to patients with high-allelic-ratio (HAR) *FLT3*-ITD (allelic ratio > 0.4) who provided additional consent for secondary enrollment	Single-institution real-world cohort; no randomization
**Induction I**	ADE 10+3+5: cytarabine 100 mg/m^2/dose q12h, d1–10; daunorubicin 50 mg/m^{2,} d1, 3, 5; etoposide 100 mg/m^{2,} d1–5; IT cytarabine, d1	ADE 10+3+5 (as Arm A) + bortezomib 1.3 mg/m^2 IV, d1, 4, 8	ADE 10+3+5 + sorafenib 200 mg/m^2 PO daily (Cohort 3: d11–28; earlier cohorts per amendment-specific schedules)	ADE 10+3+5 (same backbone and doses)
**Induction II**	LR: ADE 8+3+5 (cytarabine 100 mg/m^2/dose q12h, d1–8; daunorubicin and etoposide as above); HR: MA (cytarabine 1,000 mg/m^2/dose q12h, d1–4; mitoxantrone 12 mg/m^{2,} d3–6)	As Arm A + bortezomib 1.3 mg/m^2 IV, d1, 4, 8	As Arm A + sorafenib 200 mg/m^2 PO daily (Cohort 3: from d9 for 28 days)	LR: ADE 8+3+5; IR/HR: AM (cytarabine 8 g/m^2 cumulative + mitoxantrone)
**Intensification I**	AE: cytarabine 1,000 mg/m^2/dose q12h, d1–5; etoposide 150 mg/m^{2,} d1–5	AE + bortezomib 1.3 mg/m^2 IV, d1, 4, 8	AE + sorafenib 200 mg/m^2 PO daily (Cohort 3: from d6 for 28 days)	AE (cytarabine 10 g/m^2 cumulative + etoposide 750 mg/m^2 cumulative)
**Intensification II / SCT**	LR: cytarabine/mitoxantrone; HR: best available allogeneic SCT; if no suitable donor, HD-AraC/L-asparaginase (cytarabine 3,000 mg/m^2/dose q12h, d1, 2, 8, 9 + L-asparaginase)	As Arm A + bortezomib 1.3 mg/m^2 IV, d1, 4, 8 (each course)	As Arm A + sorafenib; sorafenib maintenance 100 mg/m^2 PO daily for 1 year, starting d40–100 after SCT or Intensification II	LR: AM (cytarabine 8 g/m^2 + mitoxantrone 48 mg/m^2 cumulative); IR/HR: allogeneic HSCT if a donor was available; otherwise AL (cytarabine 12 g/m^2 cumulative + L-asparaginase)
***FLT3* inhibitors**	None	None	Sorafenib (as above)	None administered

Abbreviations: ADE, cytarabine/daunorubicin/etoposide; AE, cytarabine/etoposide; MA/AM, cytarabine/mitoxantrone; AL, cytarabine/L-asparaginase; HD-AraC, high-dose cytarabine; IT, intrathecal; IV, intravenous; PO, oral; q12h, every 12 hours; d, day; SCT, stem cell transplantation; HAR, high allelic ratio; LR, low-risk; IR/HR, intermediate/high-risk; HR, high-risk.

Doses are shown per protocol; for patients with a body surface area < 0.6 m^2, chemotherapy was dosed in mg/kg as specified in the protocol. Within Arm C, the timing of sorafenib initiation differed across the three sequential cohorts (Cohorts 1–3) because of cardiac toxicity concerns; sorafenib was started after completion of conventional chemotherapy per Amendment #6A in Cohort 3. No patient in the CHCMU cohort received sorafenib or other *FLT3* inhibitors. Cumulative doses are reported for the CHCMU regimen as per the institutional protocol. Arm Ns denote TARGET cohort patients treated on the AAML1031 backbone with available treatment-arm annotation, who constitute the analysis set for the arm-specific survival analyses (Figure S2)

Treatment details are summarized from the Children's Oncology Group phase III trial AAML1031 (NCT01371981) and from Aplenc et al. **[9]** and Pollard et al. **[44]**; the CHCMU regimen is shown according to the local institutional protocol.
