# Supplementary material for: Genomic Determinants of Induction Failure and Persistent Prognostic Risk of FLT3-ITD in Pediatric Acute Myeloid Leukemia
Source: Cancers (Basel). 2026 Oct 12;18(20):3280. doi: 10.3390/cancers18203280 (PMC13702683; doi:10.3390/cancers18203280)
Supplement: Supplementary file 1 [file cancers-18-03280-s001.zip › Table S10.pdf]

Table S10. Extended multivariable model including cytogenetic covariates assessing whether cytogenetic risk confounds the *FLT3*–IF association in the TARGET cohort.

**Variable**	**OR (95% CI)**	***p* value**
FLT3 mutation	2.50 (1.21–5.15)	**0.013**
WT1 mutation	1.88 (0.88–4.02)	0.103
NPM1 mutation	NE†	—
KIT mutation	0.38 (0.04–3.19)	0.370
WBC (>50 × 10^9/L)	1.98 (0.99–3.95)	0.053
Female sex	0.50 (0.26–0.98)	**0.043**
BM blasts (>70%)	1.43 (0.70–2.91)	0.321
Favorable karyotype [t(8;21)/inv(16)]	0.26 (0.07–0.96)	**0.044**
Monosomy 7	8.50 (2.39–30.26)	**0.001**
Normal karyotype	1.75 (0.83–3.67)	0.139

Note: Cytogenetic covariates (favorable karyotype [t(8;21)/inv(16)], monosomy 7, and normal karyotype) were added to the multivariable model described in Table 1 (*FLT3*, *WT1*, *NPM1*, *KIT*, WBC > 50 × 10^9/L, female sex, and BM blasts > 70%). † Not estimable: no IF events occurred among *NPM1*-mutated patients in this complete-case cohort.
