# Supplementary material for: Genomic Determinants of Induction Failure and Persistent Prognostic Risk of FLT3-ITD in Pediatric Acute Myeloid Leukemia
Source: Cancers (Basel). 2026 Oct 12;18(20):3280. doi: 10.3390/cancers18203280 (PMC13702683; doi:10.3390/cancers18203280)
Supplement: Supplementary file 1 [file cancers-18-03280-s001.zip › Table S11.pdf]

Table S11A. Interaction between *NPM1* mutation and *FLT3*-ITD on OS in the TARGET cohort.

NPM1 genotype	FLT3-ITD+ (n)	HR (95% CI)	*p* value
wild-type	279	1.50 (1.24–1.82)	< 0.001
mutated	61	1.20 (0.56–2.55)	0.64

Cox models adjusted for age, sex, WBC count, and FAB subtype.

The *NPM*1–*FLT3*-ITD interaction term was not statistically significant ($p = 0.566$).

Table S11B. Interaction between *NPM1* mutation and *FLT3*-ITD on IF in the TARGET cohort.

NPM1 genotype	*FLT3*-ITD	IF (n/N)	IF rate (%)
wild-type	−	31/491	6.3
wild-type	+	23/93	24.7
mutated	−	1/44	2.3
mutated	+	0/30	0.0

IF rates by *NPM1*/*FLT3*-ITD genotype among TARGET patients with validated *NPM1* genotypes.

Fisher's exact test (two-sided): $p = 0.001$ for *NPM1*-mutated/*FLT3*-ITD-positive versus *NPM1*-WT/*FLT3*-ITD-positive patients.

Abbreviations: OS, overall survival; IF, induction failure; HR, hazard ratio.
