# Supplementary material for: Genomic Determinants of Induction Failure and Persistent Prognostic Risk of FLT3-ITD in Pediatric Acute Myeloid Leukemia
Source: Cancers (Basel). 2026 Oct 12;18(20):3280. doi: 10.3390/cancers18203280 (PMC13702683; doi:10.3390/cancers18203280)
Supplement: Supplementary file 1 [file cancers-18-03280-s001.zip › Table S12.pdf]

Table S12. Association of salvage therapy and HSCT with mutation status of key genes (*FLT3*, *WT1*, *KIT*, *NRAS*, and *CEBPA*) among the IF patients in the CHCMU cohort.

**Gene**	**Salvage, *n* (%)**	**No salvage, *n* (%)**	***p* value**	**HSCT, *n* (%)**	**No HSCT, *n* (%)**	***p* value**
FLT3	7/16 (43.8)	3/5 (60.0)	0.635	4/6 (66.7)	6/15 (40.0)	0.362
WT1	5/16 (31.2)	0/5 (0.0)	0.278	3/6 (50.0)	2/15 (13.3)	0.115
KIT	3/16 (18.8)	3/5 (60.0)	0.115	0/6 (0.0)	6/15 (40.0)	0.123
NRAS	7/16 (43.8)	1/5 (20.0)	0.607	2/6 (33.3)	6/15 (40.0)	1.000
CEBPA	2/16 (12.5)	0/5 (0.0)	1.000	1/6 (16.7)	1/15 (6.7)	0.500

Data are presented as *n* (%). Of the 21 IF patients, 16 (76.2%) received salvage chemotherapy and 6 (28.6%) underwent HSCT. *p* values from Fisher's exact test comparing patients with versus without salvage therapy (left) and patients with versus without HSCT (right). *NPM1* mutations were not detected among IF patients and were therefore not included. HSCT, hematopoietic stem cell transplantation.
