# Supplementary material for: Genomic Determinants of Induction Failure and Persistent Prognostic Risk of FLT3-ITD in Pediatric Acute Myeloid Leukemia
Source: Cancers (Basel). 2026 Oct 12;18(20):3280. doi: 10.3390/cancers18203280 (PMC13702683; doi:10.3390/cancers18203280)
Supplement: Supplementary file 1 [file cancers-18-03280-s001.zip › Table S2.pdf]

**Table S2A. Baseline characteristics of pAML patients in the TARGET cohort**

| Indicators | | IF(N=171) N(%) | non-IF(N=1808) N(%) | *P* |
|---|---|---|---|---|
| **Gender** | | | | **0.013** |
| | Female | 66(38.6%) | 881(48.7%) | |
| | Male | 105(61.4%) | 927(51.3%) | |
| **Age at diagnosis** | | | | **0.020** |
| | P50 (P25, P75) | 7.8(2.4-13.4) | 10.3(3.4-15.1) | |
| | <3 | 49(28.7%) | 410(22.7%) | |
| | 3–10 | 53(31.0%) | 468(25.9%) | |
| | ＞10 | 69(40.4%) | 930(51.4%) | |
| **WBC at Diagnosis(×10^9/L)** | | | | **0.000** |
| | P50 (P25, P75) | 46.8(13.4-165.2) | 25.0(8.3-75.9) | |
| | 0–50 | 86(50.3%) | 1174(64.9%) | |
| | 50–100 | 25(14.6%) | 289(16.0%) | |
| | ≥100 | 60(35.1%) | 345(19.1%) | |
| **FAB classification** | | | | 0.355 |
| | M0 | 3(1.8%) | 19(1.1%) | |
| | M1 | 13(7.6%) | 88(4.9%) | |
| | M2 | 14(8.2%) | 190(10.5%) | |
| | M4 | 17(9.9%) | 192(10.6%) | |
| | M5 | 15(8.8%) | 155(8.6%) | |
| | M6 | 0(0.0%) | 13(0.7%) | |
| | M7 | 5(2.9%) | 36(2.0%) | |
| | other | 7(4.1%) | 79(4.3%) | |
| | unknown | 97(56.7%) | 1036(57.3%) | |
| **CNS disease** | | | | 0.402 |
| | Yes | 89(52.0%) | 978(54.1%) | |
| | No | 80(46.8%) | 780(43.1%) | |
| | NA | 2(1.2%) | 50(2.8%) | |
| **Protocol** | | | | 0.600 |
| | AAML1031 | 84(49.1%) | 977(54.0%) | |
| | AAML03P1 | 10(5.8%) | 88(4.9%) | |
| | AAML0531 | 70(40.9%) | 687(38.0%) | |
| | CCG2961 | 7(4.1%) | 56(3.1%) | |
| **BM blasts** | | | | **0.024** |
| | P50 (P25, P75) | 76(51-88) | 69(43-85) | |
| **Peripheral blasts** | | | | **0.029** |
| | P50 (P25, P75) | 52(17-79.5) | 41(12-71) | |
| **Risk group** | | | | **0.000** |
| High | | 51(29.8%) | 207(11.4%) | |
| Low | | 15(8.8%) | 709(39.2%) | |
| Standard | | 101(59.1%) | 837(46.3%) | |

| | | | |
|---|---|---|---|
| Unknown | 3(1.7%) | 30(1.7%) | |
| NA | 1(0.6%) | 25(1.4%) | |

**Table S2B. Baseline characteristics of pAML patients in the CHCMU cohort**

| **Indicators** | | **IF(N=21) N(%)** | **non-IF(N=77) N(%)** | ***p*** |
|---|---|---|---|---|
| **Gender** | | | | |
| | | | | 0.323 |
| | Female | 7(33.3%) | 37(48.1%) | |
| | Male | 14(66.7%) | 40(51.9%) | |
| **Age at diagnosis** | | | | |
| | P50 (P25, P75) | 9.6(5.1-11.7) | 4.7(2.8-12.0) | 0.7 |
| | <3 | 4(19.0%) | 18(23.4%) | |
| | 3–10 | 8(38.1%) | 34(44.2%) | |
| | 10–18 | 9(42.9%) | 25(32.5%) | |
| **CNSL** | | | | 0.854 |
| | CNS1 | 19(90.5%) | 62(80.5%) | |
| | CNS2 | 0(0.0%) | 4(5.2%) | |
| | CNS3 | 0(0.0%) | 2(2.6%) | |
| | NA | 2(9.5%) | 9(11.7%) | |
| **WBC at diagnosis (×10^9/L)** | | | | 0.999 |
| | P50 (P25, P75) | 14.37 (8.66, 53.44) | 15.56 (5.92, 51.85) | |
| | 0–50 | 16(76.2%) | 59(76.6%) | |
| | 50–100 | 3(14.3%) | 10(13.0%) | |
| | ≥100 | 2(9.5%) | 8(10.4%) | |
| **FAB classification** | | | | 0.861 |
| | M1 | 0(0.0%) | 5(6.5%) | |
| | M2 | 12(57.1%) | 38(49.4%) | |
| | M4 | 2(9.5%) | 8(10.4%) | |
| | M5 | 4(19.0%) | 16(20.8%) | |
| | M6 | 0(0.0%) | 1(1.3%) | |
| | M7 | 1(4.8%) | 6(7.8%) | |
| | other | 2(9.5%) | 3(3.9%) | |
| **Chromosome karyotype** | | | | 0.565 |
| | normal | 8(38.1%) | 22(28.6%) | |
| | abnormal | 11(52.4%) | 50(64.9%) | |
| | NA | 2(9.5%) | 5(6.5%) | |
| **Risk stratification at initial diagnosis** | | | | 0.852 |
| | LR | 2(9.5%) | 13(16.9%) | |
| | IR | 7(33.3%) | 25(32.5%) | |
| | HR | 11(52.4%) | 36(46.8%) | |

| | | | | |
|---|---|---|---|---|
| | NA | 1(4.8%) | 3(3.9%) | |
| **Peripheral blasts** | | | | |
| | P50 (P25, P75) | 33.00 (21.25, 60.50) | 44.00 (29.00, 61.00) | 0.302 |
| **BM blasts** | | | | **0.004** |
| | P50 (P25, P75) | 60.45 (28.85, 79.30) | 44.00 (29.00, 61.00) | |
| **HSCT** | | | | 0.999 |
| | Yes | 7(33.3%) | 28(36.4%) | |
| | No | 14(66.7%) | 49(63.6%) | |

Note: WBC: White blood cell; CNSL: Central nervous system leukemia;HSCT: Hematopoietic Stem Cell Transplantation;BM blasts, Bone marrow leukemic blast percentage; Karyotype was classified as normal (46,XX/46,XY) or abnormal based on conventional cytogenetic analysis.
