# Supplementary material for: Genomic Determinants of Induction Failure and Persistent Prognostic Risk of FLT3-ITD in Pediatric Acute Myeloid Leukemia
Source: Cancers (Basel). 2026 Oct 12;18(20):3280. doi: 10.3390/cancers18203280 (PMC13702683; doi:10.3390/cancers18203280)
Supplement: Supplementary file 1 [file cancers-18-03280-s001.zip › Table S3.pdf]

Table S3. Time-dependent hazard ratios of IF for OS

Time period	HR (95% CI)	*p* value	N	Events
0-6 months	5.57 (3.58–8.66)	<0.001	1979	90
6-24 months	3.29 (2.57–4.23)	<0.001	1862	455
>24 months	1.59 (0.81–3.12)	0.182	1352	133

NOTE: Multivariable Cox regression models stratified by time periods (0–6, 6–24, and >24 months) were adjusted for age, sex, WBC count, and FAB subtype (grouped as M0, M1/M2, M4/M5, M6/M7, and Other). HR, hazard ratio; CI, confidence interval; IF, induction failure. N represents the number of patients at risk at the beginning of each time interval; Events represents the number of deaths observed within each interval.
