# Supplementary material for: Genomic Determinants of Induction Failure and Persistent Prognostic Risk of FLT3-ITD in Pediatric Acute Myeloid Leukemia
Source: Cancers (Basel). 2026 Oct 12;18(20):3280. doi: 10.3390/cancers18203280 (PMC13702683; doi:10.3390/cancers18203280)
Supplement: Supplementary file 1 [file cancers-18-03280-s001.zip › Table S4.pdf]

Table S4. Clinical characteristics of patients with pathogenic mutations (Levels 1 and 2) in the CHCMU validation cohort

Mutation gene	N	Age, years Median (IQR)	Male, *n*	FAB subtype, n	IF, *n*	non-IF, *n*	Level
NRAS	23	5.28 (2.78, 9.60)	9	M1(1), M2(11), M4(6), M5(3), other(2)	8	15	1
KIT	19	9.46 (3.85, 11.82)	13	M2(16), M4(3)	5	14	1
FLT3	17	9.69 (5.35, 12.35)	9	M1(2), M2(8), M4(3), M5(2), other(2)	10	7	1
WT1	11	7.45 (4.26, 11.78)	6	M1(2), M2(5), M4(1), M5(2), other(1)	4	7	1
CEBPA	10	7.67 (3.86, 8.34)	3	M1(2), M2(8)	1	9	1
KRAS	10	2.59 (1.69, 4.25)	6	M2(2), M4(2), M5(6)	1	9	1
PTPN11	4	3.68 (1.78, 7.42)	4	M2(1), M5(2), M6(1)	1	3	1
ASXL1	2	1.69, 6.27	2	M5(2)	1	1	1
NPM1	2	9.69	0	M1(1), other(1)	0	2	1
IDH2	1	9.69	0	M1(1)	0	1	1
RUNX1	1	12.60	1	other(1)	1	0	1
TP53	1	6.27	1	M5(1)	1	0	1
ASXL2	11	4.69 (3.96, 8.52)	6	M2(11)	2	9	2
FLT3	5	12.34 (4.02, 12.60)	2	M2(2), M5(2), other(1)	1	4	2
CEBPA	4	12.81 (9.58, 14.15)	0	M1(2), M2(1), other(1)	1	3	2
CSF3R	4	7.03 (5.40, 9.32)	2	M2(4)	1	3	2
GATA2	3	7.67	1	M2(1), M5(1), other(1)	0	3	2
KDM6A	3	4.93 (4.81, 8.69)	2	M2(1), M5(2)	1	2	2
PHF6	3	13.69, 15.54	1	M1(1), M2(2)	0	3	2
CBL	2	0.08, 5.25	2	M2(2)	0	2	2
CCND2	2	3.87	2	M2(2)	0	2	2
CTCF	2	4.62, 12.51	1	M2(2)	0	2	2
DHX15	2	3.69, 12.33	1	M2(2)	0	2	2
EP300	2	12.60	2	M2(1), other(1)	1	1	2
ETV6	2	2.59, 5.44	1	M2(1), M6(1)	0	2	2
EZH2	2	5.95, 12.51	1	M2(2)	0	2	2
JAK2	2	14.45	2	M2(1), M7(1)	0	2	2
JAK3	2	2.49, 4.26	0	M2(1), other(1)	1	1	2
KMT2D	2	11.68, 12.45	1	M2(1), M5(1)	1	1	2
ARID2	1	2.78	1	M4(1)	0	1	2
ATM	1	8.50	0	M2(1)	0	1	2
ATRX	1	10.08	0	other(1)	0	1	2
BRAF	1	3.86	1	M2(1)	0	1	2
BRCA2	1	5.44	1	M6(1)	0	1	2
BUB1B	1	2.60	1	M4(1)	0	1	2
DIS3	1	3.69	0	M2(1)	0	1	2
DNM2	1	3.83	1	M2(1)	0	1	2
DNMT3A	1	3.63	0	M5(1)	0	1	2
FBXO11	1	5.42	1	M2(1)	1	0	2
IKZF1	1	2.59	0	M2(1)	0	1	2
KIT	1	5.25	1	M2(1)	0	1	2
MAP3K1	1	3.69	0	M2(1)	0	1	2
MGA	1	5.95	0	M2(1)	0	1	2
MSH6	1	8.34	0	M1(1)	0	1	2
MYC	1	8.50	0	M2(1)	0	1	2
NF1	1	12.36	1	M4(1)	1	0	2
NSD1	1	4.26	0	M2(1)	0	1	2
PCBP1	1	10.42	1	M2(1)	0	1	2
PTEN	1	3.83	1	M2(1)	0	1	2
RAD21	1	9.60	0	M2(1)	1	0	2
SETD2	1	7.43	0	M5(1)	1	0	2
SMC1A	1	4.34	1	M5(1)	0	1	2
STAG2	1	5.25	1	M2(1)	0	1	2
TP53	1	2.11	0	M2(1)	0	1	2
U2AF1	1	12.51	1	M2(1)	0	1	2
UBTF	1	13.34	1	M2(1)	1	0	2
XPO1	1	0.26	1	M5(1)	0	1	2
XRCC2	1	8.34	0	M1(1)	0	1	2

Note: Mutation counts are based on unique patients (each patient counted once per gene per level).

Abbreviations: IQR, interquartile range; FAB, French-American-British classification; IF, induction failure.
