# Supplementary material for: Genomic Determinants of Induction Failure and Persistent Prognostic Risk of FLT3-ITD in Pediatric Acute Myeloid Leukemia
Source: Cancers (Basel). 2026 Oct 12;18(20):3280. doi: 10.3390/cancers18203280 (PMC13702683; doi:10.3390/cancers18203280)
Supplement: Supplementary file 1 [file cancers-18-03280-s001.zip › Table S6.pdf]

Table S6. Clinical characteristics of patients with fusion genes in the CHCMU validation cohort.

Fusion Gene	N	Age, years Median (IQR)	Male, *n*	FAB subtype, *n*	IF, *n*	non-IF, *n*	level
RUNX1-RUNX1T1	26	5.42 (3.85-10.62)	17	M2(26)	4	22	1
CBFB-MYH11	9	12.02 (7.45-12.36)	5	M2(2), M4(6), other(1)	2	7	1
KMT2A-MLLT10	8	3.52 (1.53-7.14)	5	M2(1), M4(1), M5(6)	2	6	1
KMT2A-MLLT3	5	4.02 (2.76-12.31)	1	M5(5)	1	4	1
NUP98-KDM5A	2	7.20 (2.72, 11.68)	2	M2(1), M7(1)	1	1	1
CBFA2T3-GLIS2	2	1.22 (0.83, 1.61)	0	M7(2)	0	2	1
KMT2A-FLNA	1	1.69	1	M5(1)	0	1	1
KMT2A-MLLT4	1	12.45	0	M5(1)	0	1	1
KMT2A-ELL	1	4.67	0	M2(1)	1	0	1
KMT2A-MLLT6	1	2.17	1	other(1)	0	1	1
KMT2A-MLLT11	1	0.83	0	M2(1)	1	0	1
KMT2A-SEPT6	1	3.86	1	M2(1)	0	1	1
KAT6A-CREBBP	1	0.26	1	M5(1)	0	1	1
FUS-ERG	4	3.23 (1.97, 10.80)	2	M2(2), M4(1), M5(1)	0	4	2
NUP98-NSD1	1	2.08	0	M4(1)	0	1	2
CBFA2T3-GLIS2	1	7.02	1	M7(1)	0	1	2
DDX3X-MLLT10	1	13.67	0	M1(1)	0	1	2
TBC1D15-RAB21	1	3.60	0	M2(1)	0	1	2
ZEB2-BCL11B	1	8.08	1	M5(1)	0	1	2
RUNX1T1-RUNX1	8	4.48 (2.90, 12.15)	6	M2(8)	1	7	3
ZNF292-SYNCRIP	2	6.19 (1.61, 10.78)	2	M4(1), M5(1)	0	2	3
RMND5A-ANAPC1	2	1.30 (0.99, 1.61)	2	M4(1), M7(1)	0	2	3
PSPC1-ZMYM2	2	1.47 (0.82, 2.11)	0	M2(1), M7(1)	0	2	3
MBP-ZNF516	2	0.99	1	M7(1)	0	2	3
KDM5A-NUP98	2	7.20 (2.72, 11.68)	2	M2(1), M7(1)	1	1	3
ERG-FUS	2	8.65 (4.34, 12.95)	1	M4(1), M5(1)	0	2	3
CBFB-MYH11	2	1.62	1	M4(2)	0	2	3

FLNA-KMT2A	1	1.69	1	M5(1)	0	1	3
MLLT3-KMT2A	1	3.63	0	M5(1)	0	1	3
C16orf72-USP7	1	4.7	1	M2(1)	0	1	3
ELP4-EHF	1	4.26	0	M2(1)	0	1	3
FKBP8-CCDC84	1	4.67	0	M2(1)	1	0	3
IGKV1-27-ZCCHC7	1	8.86	0	M2(1)	0	1	3
HNRNPAB-AHRR	1	8.86	0	M2(1)	0	1	3
CCDC174-TTC12	1	0.82	0	M5(1)	0	1	3
MLLT10-TBC1D5	1	0.82	0	M5(1)	0	1	3
MLLT10-ZNF202	1	1.35	1	M5(1)	0	1	3
MLLT6-KMT2A	1	2.17	0	M4(1)	0	1	3
NSD1-NUP98	1	2.04	0	M5(1)	0	1	3
TUBGCP6-MAPK12	1	3.87	1	M2(1)	0	1	3
MLLT10-UVRAG	1	13.69	0	M1(1)	0	1	3
DESI2-KIF26B	1	13.69	0	M1(1)	0	1	3
MLLT10-RGAG4	1	12.81	1	M2(1)	0	1	3
PARG-BMS1	1	12.81	1	M2(1)	0	1	3
PTBP1-PALM	1	3.50	1	M5(1)	0	1	3
CCNY-TLK1	1	7.43	0	M5(1)	1	0	3
MLLT10-CCNY	1	7.43	0	M5(1)	1	0	3
ATP5L-KMT2A	1	3.50	1	M5(1)	0	1	3
PKD1-NPIPA5	1	10.78	1	M5(1)	0	1	3
C15orf57-CBX3	1	10.78	1	M5(1)	0	1	3
SUZ12P-NF1	1	12.36	1	M4(1)	1	0	3
SYNE1-ESR1	1	13.34	1	M2(1)	1	0	3
CTNNBL1-EIF4G3	1	13.84	0	M5(1)	0	1	3
SGSM3-ADSL	1	16.53	0	M2(1)	0	1	3
MLLT10-KMT2A	1	4.25	0	M5(1)	0	1	3
KMT2A-SIK3	1	4.25	0	M5(1)	0	1	3
SIK3-KMT2A	1	4.25	0	M5(1)	0	1	3
KLF10-YWHAZ	1	1.62	1	M4(1)	0	1	3
OAZ1-ZBTB7A	1	1.62	1	M4(1)	0	1	3
OAZ1-CSNK1G2	1	0.99	1	M7(1)	0	1	3
OAZ1-DAZAP1	1	0.99	1	M7(1)	0	1	3
