# Supplementary material for: Genomic Determinants of Induction Failure and Persistent Prognostic Risk of FLT3-ITD in Pediatric Acute Myeloid Leukemia
Source: Cancers (Basel). 2026 Oct 12;18(20):3280. doi: 10.3390/cancers18203280 (PMC13702683; doi:10.3390/cancers18203280)
Supplement: Supplementary file 1 [file cancers-18-03280-s001.zip › Table S8.pdf]

Table S8. Univariate comparison of cytogenetic aberrations between IF and non-IF patients in the TARGET cohort.

**Cytogenetic feature**	**IF (*n* = 55), *n* (%)**	**non-IF (*n* = 604), *n* (%)**	**OR (95% CI)**	***p* value**
t(8;21)	3 (5.5)	98 (16.2)	0.34 (0.11–1.03)	**0.031**
inv(16)	1 (1.8)	96 (15.9)	0.11 (0.02–0.80)	**0.002**
KMT2A rearrangements	9 (16.4)	108 (17.9)	0.92 (0.49–1.70)	1.00
Monosomy 7 (-7)	5 (9.1)	10 (1.7)	5.49 (1.95–15.50)	**0.005**
Normal karyotype	20 (36.4)	143 (23.7)	1.54 (1.05–2.24)	**0.049**
+8	6 (10.9)	50 (8.3)	1.32 (0.59–2.93)	0.45
del(7q)	2 (3.6)	13 (2.2)	1.69 (0.39–7.30)	0.36
Other cytogenetic abnormalities	18 (32.7)	137 (22.7)	1.44 (0.96–2.17)	0.098

Note: Frequencies were compared by Fisher's exact tests among 659 TARGET patients with available karyotypes. Favorable-risk core-binding factor karyotypes (t(8;21) or inv(16)) were present in 4 (7.3%) IF versus 188 (31.1%) non-IF patients (OR = 0.17, 95% CI: 0.06–0.49, $p < 0.001$). OR, odds ratio.
