# Supplementary material for: Genomic Determinants of Induction Failure and Persistent Prognostic Risk of FLT3-ITD in Pediatric Acute Myeloid Leukemia
Source: Cancers (Basel). 2026 Oct 12;18(20):3280. doi: 10.3390/cancers18203280 (PMC13702683; doi:10.3390/cancers18203280)
Supplement: Supplementary file 1 [file cancers-18-03280-s001.zip › Table S9.pdf]

Table S9. Multivariable logistic regression of cytogenetic and clinical factors associated with IF in the TARGET cohort.

Variable	OR (95% CI)	*p* value
Age (per year)	0.98 (0.93–1.03)	0.41
Male sex	1.14 (0.63–2.05)	0.67
WBC count (per 10^9/L)	1.01 (1.00–1.01)	**< 0.001**
t(8;21)	0.38 (0.11–1.36)	0.14
inv(16)	0.09 (0.01–0.69)	**0.02**
KMT2A rearrangement	0.65 (0.27–1.59)	0.35
Normal karyotype	1.47 (0.72–2.99)	0.29
Monosomy 7	6.13 (1.78–21.05)	**0.004**

Note: Model adjusted for age, sex, WBC count, and cytogenetic features with $p < 0.10$ in univariate analyses.
